# Spatiotemporal segregation of human marginal zone and memory B cell populations in lymphoid tissue

**DOI:** 10.1038/s41467-018-06089-1

**Published:** 2018-09-21

**Authors:** Yuan Zhao, Mohamed Uduman, Jacqueline H. Y. Siu, Thomas J. Tull, Jeremy D. Sanderson, Yu-Chang Bryan Wu, Julian Q. Zhou, Nedyalko Petrov, Richard Ellis, Katrina Todd, Konstantia-Maria Chavele, William Guesdon, Anna Vossenkamper, Wayel Jassem, David P. D’Cruz, David J. Fear, Susan John, Dagmar Scheel-Toellner, Claire Hopkins, Estefania Moreno, Natalie L. Woodman, Francesca Ciccarelli, Susanne Heck, Steven H. Kleinstein, Mats Bemark, Jo Spencer

**Affiliations:** 1 School of Immunology and Microbial Sciences, King’s College London, Guy’s Campus, London, SE1 9RT UK; 2 Department of Pathology, Yale University School of Medicine, New Haven, CT 06511 USA; 3 Department of Surgery, University of Cambridge, Cambridge, CB2 0QQ UK; 4 Randall Division of Cell and Molecular Biophysics, King’s College London, London, SE1 1UL UK; 5 Interdepartmental Program in Computational Biology and Bioinformatics, Yale University, New Haven, CT 06511 USA; 6 Biomedical Research Centre, Guy’s and St. Thomas’ NHS Trust, London, SE1 9RT UK; 7 Barts & The London School of Medicine and Dentistry, Blizard Institute, Whitechapel, London, E1 2AT UK; 8 Liver Transplant Unit, Institute of Liver Studies, King’s College Hospital, Denmark Hill, London, SE5 9NT UK; 9 Institute of Inflammation and Ageing, College of Medical and Dental Sciences, University of Birmingham, Birmingham, B15 2TT UK; 10 School of Cancer Sciences, King’s College London, Guy’s Campus, London, SE1 9RT UK; 11 Department of Immunobiology, Yale University School of Medicine, New Haven, CT 06511 USA; 12 Mucosal Immunobiology and Vaccine Center (MIVAC), Department of Microbiology and Immunology, Institute of Biomedicine, Sahlgrenska Academy, University of Gothenburg, SE 405 30 Gothenburg, Sweden

## Abstract

Human memory B cells and marginal zone (MZ) B cells share common features such as the expression of CD27 and somatic mutations in their *IGHV* and *BCL6* genes, but the relationship between them is controversial. Here, we show phenotypic progression within lymphoid tissues as MZ B cells emerge from the mature naïve B cell pool via a precursor CD27^−^CD45RB^MEM55+^ population distant from memory cells. By imaging mass cytometry, we find that MZ B cells and memory B cells occupy different microanatomical niches in organised gut lymphoid tissues. Both populations disseminate widely between distant lymphoid tissues and blood, and both diversify their IGHV repertoire in gut germinal centres (GC), but nevertheless remain largely clonally separate. MZ B cells are therefore not developmentally contiguous with or analogous to classical memory B cells despite their shared ability to transit through GC, where somatic mutations are acquired.

## Abstract (web summary)

Human memory and marginal zone B cells share some features including CD27 expression and somatic hypermutation, but their lineage relationship is still unclear. Here the authors use mass cytometry and sequential clustering methods to show that, despite their shared features, memory and marginal zone B cells represent distinct lineage choices.

## Introduction

Marginal zone (MZ) B cells are CD27^+^IgM^+^IgD^+^ cells that occupy the microanatomical niche on the periphery of the white pulp in the spleen of humans^1–3^. They make innate-like responses to T independent antigens and are major contributors to systemic anti-bacterial immunity^3,4^. Phenotypically similar cells in blood are often referred to as circulating MZ B cells^5^. The populations share many properties with memory B cells that provide high affinity antibody responses to recall antigens, including expression of CD27 and somatic mutations in Ig V regions and *BCL6* that are classically acquired during germinal centre (GC) responses^6,7^. Despite these similarities, the prevailing hypothesis is that MZ B cells are not *bona fide* memory B cells but rather a separate lineage derived from CD27^−^ precursors that express the CD45RB^MEM55^ epitope (CD45RB^+^) through ligation of NOTCH2 in spleen^8,9^. It has been suggested that they may acquire somatic mutations independent of GC^1,10^. This scheme, whilst supported by studies of blood and spleen from children^9^; emergence of B cells following haematopoietic stem cell transplantation^8^; and the presence of such cells in patients lacking CD40 signalling^10^, is yet to gain general acceptance and cannot explain where MZ B cells proliferate to acquire somatic mutations in their *IGV* genes.

In healthy humans, both spleen and gut-associated lymphoid tissue (GALT) have microanatomically defined MZs^11^. These sites may share a lymphocyte pool as their endothelial and reticular structures express MAdCAM1 that have the potential to recruit cells expressing α4β7 integrin^12^. Thus, circulating MZ-like B cells could be in transit between spleen and GALT. In support of shared B cell populations, self-reactive IgM-expressing MZ B cell lymphomas of mucosa-associated lymphoid tissue can metastasise to the splenic MZ^13^. Moreover, GALT is required for the development of the splenic marginal zone in rabbits^14^. Whilst a recent study proposed that the IgM-expressing CD27^+^ cells in GALT are memory B cells rather than MZ B cells, it did not consider the possibility that MZ B cells characterised by their IgD expression may also be present, and made conclusions based on the properties of CD27^+^IgM^+^IgD^−^ cells (IgM-only cells) from the gut^15^.

To resolve these issues, we visualise phenotypic progression in B cells isolated from human GALT, spleen and tonsils by mass cytometry. We observe that MZ B cells in these tissues are phenotypically linked to a CD27^−^CD45RB^+^ precursor population, forming a separate developmental branch from the naïve B cell pool that was phenotypically separate from that of memory B cells. Imaging mass cytometry is used to localise MZ B cells in GALT. This demonstrates that MZ B cells and memory B cells reside in different microanatomical niches. Whereas memory B cells occupy the lymphoid tissue boundaries, MZ B cells have a more limited distribution adjacent to the GC. Finally, we use *IGHV* gene analysis to identify that both MZ B cells and memory B cells disseminate between distant sites of GALT and circulate in the blood, and that both can diversify their *IGHV* genes in the GC of GALT whilst remaining clonally separate. We conclude that human MZ B cells develop separately from classical memory B cells and that their archetypical mutations are likely introduced as they proliferate in GALT GC.

## Results

### Visualisation of B cells from tissues by mass cytometry

**Fig. 1:**
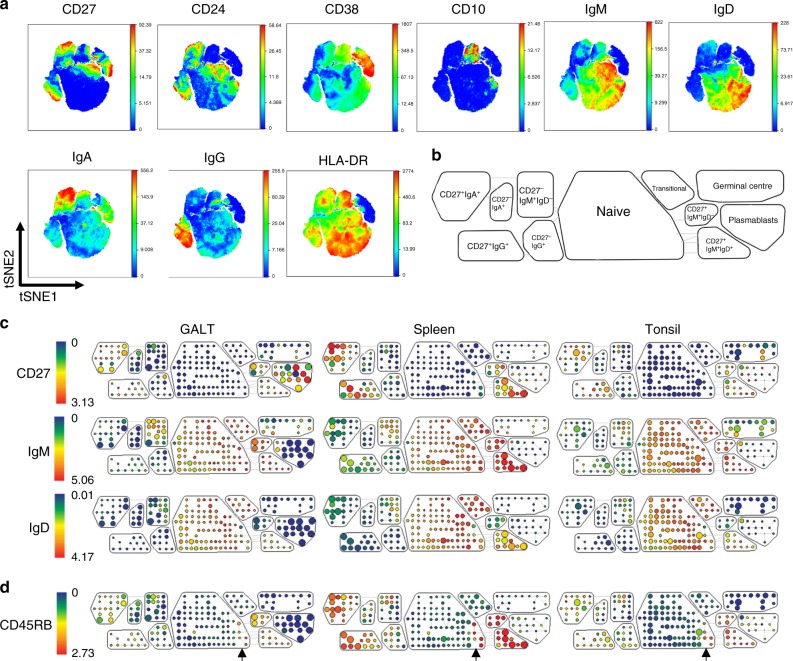
Deep immunophenotypic profiling of B cells in human tissues. **a** viSNE projections of the B cell compartment in tissues (GALT *n* = 8; tonsil *n* = 5, spleen *n* = 6). Data were clustered according to criteria used to define known B cell subsets: CD27, CD24, CD28, CD10, IgM, IgD, IgA, IgG and HLA-DR. **b** Key to B cells subsets identified in the SPADE plots in **c** that were created for each tissue to enable comparisons of their compositions. Expression of CD27, IgM and IgD are illustrated. **d** Visualisation of the expression of CD45RB in B cell subsets when CD45RB was not one of the clustering criteria. Arrows identify the location of CD45RB^+^ cells in the naïve B cell SPADE bubble

For an in-depth comparison of B cell variability within and between human lymphoid tissues, we undertook deep phenotypic profiling by mass cytometry of cell suspensions prepared from GALT, tonsil and spleen and using a panel of 35 markers of B cell subset identity, migratory capacity and function (Supplementary Table 1). Following normalisation, quality control and gating on single CD45^+^CD3^−^CD14^−^CD19^+^ B cells, multidimensional scaling of samples identified that biological replicates of each tissue separately clustered together^16^ (Supplementary Fig. 1, Supplementary Fig. 2). Data from each tissue were concatenated, and viSNE plots demonstrated expression of subset markers CD10, CD24, CD27, CD38, IgM, IgA, IgG, IgD and HLA-DR on subpopulations of B cells (Fig. 1a, Supplementary Fig. 3). SPADE trees were individually constructed from the viSNE analysis of the different tissues, and 11 major B cell subsets were identified and enclosed in SPADE bubbles (Fig. 1b, c, Supplementary Fig. 4). These included a novel CD27^−^IgM^+^IgD^−^ subset that was phenotypically similar to IgA expressing memory cells. The memory cell subsets were phenotypically distant from the CD27^+^IgM^+^IgD^+^ MZ population, and CD27^+^IgM^+^IgD^−^ (IgM-only) cells were phenotypically more similar to CD27^+^IgM^+^IgD^+^ cells than cells within the memory cell compartments.

### Phenotypic alignment of CD27^+^IgM^+^IgD^+^ cells and precursors

**Fig. 2:**
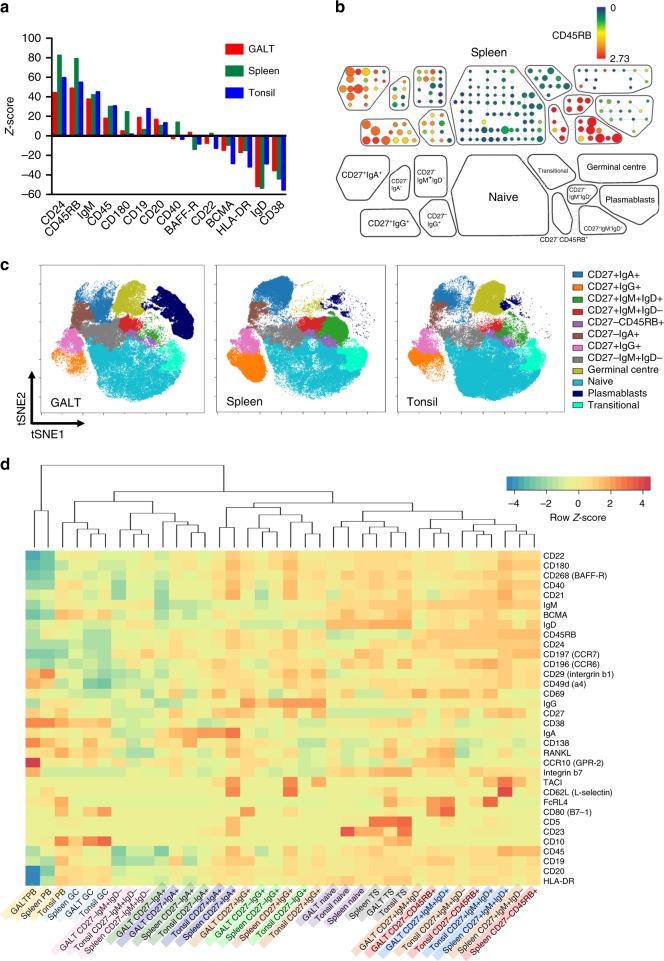
CD27^−^IgM^+^IgD^+^CD45RB^+^ marginal zone precursor B cells emerge from the naïve B cell pool. Analysis of B cells in GALT (*n* = 8); tonsil (*n* = 5), spleen (*n* = 6). **a** *Z*-score outcomes of a permutation test to determine if cell subpopulations significantly neighbour each other more than by random chance. This revealed that the CD45RB^+^ cells were significantly different to the CD45RB^-^ cells within the naïve B cell SPADE bubble by their over expression of CD24, IgM, CD45 in addition to CD45RB and lower expression of BCMA, HLA-DR, IgD and CD38. **b** A SPADE bubble was therefore placed around the CD27^−^CD45RB^+^ cells to be considered as a separate subset for inclusion in subsequent analysis, as illustrated for spleen. **c** All subsets (GC germinal centre, PB plasmablasts, TS transitional cells) were then visualised back on the viSNE plots for comparison where CD27^−^IgM^+^IgD^+^CD45RB^+^ cells were intermediate between naïve and CD27^+^IgM^+^IgD^+^ marginal zone subsets. **d** A separate hierarchical clustering method that considered all markers used for mass cytometry aligned CD27^−^IgM^+^IgD^+^CD45RB^+^ cells with CD27^+^IgM^+^IgD^+^ marginal zone cells and placed both closer to the naïve and transitional B cell subsets than conventional memory compartments

As expected, CD45RB was expressed by CD27^+^ and CD27^−^ cells^17^. Consistent with the proposed NOTCH2-dependent developmental sequence, CD27^−^CD45RB^+^ cells in the naïve B cell SPADE bubble were positioned adjacent to CD27^+^IgM^+^IgD^+^ cells in all tissues (Fig. 1d)^8,9^. Importantly, this occurred despite the fact that we did not include CD45RB expression in the viSNE criteria (Fig. 1a, c). A bootstrapping procedure to test whether cell subpopulations significantly differ from each other revealed that the CD45RB^+^ cells were significantly different to the CD45RB^−^ cells within the naïve B cell SPADE bubble^18–20^. Most notably CD27^−^IgM^+^IgD^+^CD45RB^+^ cells consistently expressed significantly higher levels of CD24 and IgM and lower IgD than other CD27^−^IgM^+^IgD^+^CD45RB^−^ naïve cells across all tissues (Fig. 2a). A separate SPADE bubble was created around the CD27^−^IgM^+^IgD^+^CD45RB^+^ cells that was designated as an independent population in subsequent analysis (Fig. 2b). When the sizes and spatial locations of the 12 subsets in tissues were visualised back on the viSNE maps, the CD27^−^IgM^+^IgD^+^CD45RB^+^ cells were positioned between CD27^+^IgM^+^IgD^+^ and naïve cells (Fig. 2c).

Having observed phenotypic alignment of CD27^+^IgM^+^IgD^+^ and their CD27^−^CD45RB^+^ precursor population according to B cell subset parameters, we undertook a separate hierarchical clustering of the 12 B cell subsets from all three tissues based on all 34 parameters measured^21^. We observed that in each tissue CD27^+^IgM^+^IgD^+^ cells were significantly aligned to their CD27^−^ CD45RB^+^ precursor population, and that this cluster was positioned closer to naïve and transitional cells than to class switched memory cells (Fig. 2d).

### A B cell subset may be different in different tissues

**Fig. 3:**
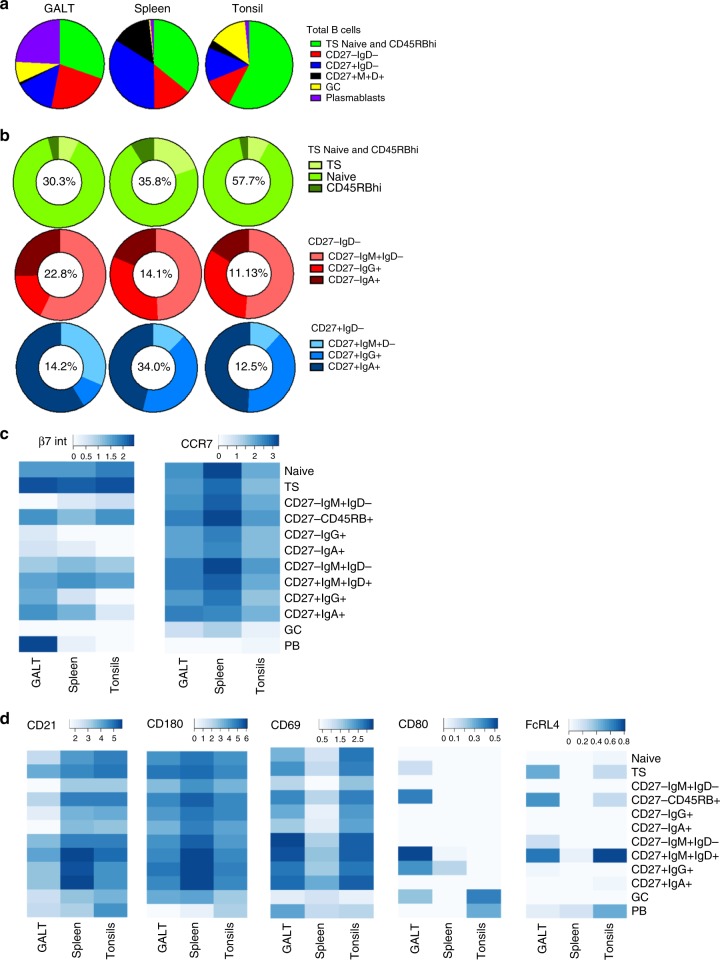
Comparison of B cell subsets between tissues. Analysis of B cells in GALT (*n* = 8); tonsil (*n* = 5), spleen (*n* = 6). **a**, **b** Pie charts illustrate that tissues contained different relative frequencies of B cell subsets. The numbers inside the donut charts are the percentages of total B cells in that tissue represented in the donut. **c** Heatmap of median expression of β7 integrin and CCR7 in SPADE bubbles from each tissue indicating biases in migratory potential. **d** Heatmap of median expression of CD21, CD180, CD69, CD80 and FcRL4 showing that the same subset in different tissues can differ markedly in its surface antigen expression

The relative proportions of subsets in tissues were consistent with current concepts of tissue structure and composition, including larger GC compartments in GALT and tonsil than spleen and a larger CD27^+^IgM^+^IgD^+^ MZ population in spleen than either other tissue (Fig. 3a, b). The relatively large proportion of CD27^+^IgM^+^IgD^−^ observed in GALT compared to spleen and tonsils in SPADE plots was also evident here (Fig. 3b). Further analysis of median expression of markers of interest within B cell subsets revealed that B cells from each tissue expressed different profiles of molecules that mediate and direct cell traffic, indicating that these may have facilitated entry or have been imprinted following entry (Fig. 3c). In addition, splenic B cell subsets generally had relatively high expression of CD21 and CD180 compared to equivalent subsets in tonsil, and CD27^+^IgM^+^IgD^+^ B cells from GALT and tonsil as well as CD27^−^CD45RB^+^ cells from tonsil expressed higher levels of CD69 and FcRL4 compared to subset counterparts isolated from spleen. In GALT, CD27^+^IgM^+^IgD^+^ B cells and and CD27^−^CD45RB^+^ also had relatively high expression of CD80 compared to the same subset in other tissues (Fig. 3d).

### GALT MZ and class switched cells have different locations

The markedly different expression of the normally epithelial-associated molecules CD80 and FcRL4 by CD27^+^IgM^+^IgD^+^ compared to CD27^+^IgM^−^ B cells in GALT suggests that these populations may occupy different microanatomical niches^22–25^. To test this, we used imaging mass cytometry to determine the distribution of B cell subsets in GALT in three samples of human appendix and tonsil^26,27^. Glass mounted tissue sections were stained with lanthanide conjugated CD19, CD20, CD10, CD27, CD24, CD38, CD45RB, IgM, IgD, CD86, CD3, CD45 and cytokeratin (Supplementary Table 2). A high-powered UV laser then ablated the tissue spot by spot with a resolution of 1000 nm. The resulting cloud of particles was transported into the argon plasma of a Helios instrument (Fluidigm) where they were atomised and ionised. The metal-isotope ion content, and, by inference, epitope abundances and distributions, were determined in a time-of-flight mass analyser. Each laser shot by this method is akin to a pixel of a tissue image which can be recomposed to a complete image. This ground-breaking technology allows high multiplexing of antigen staining in histology samples and subsequent high dimensional data analsyis.

**Fig. 4:**
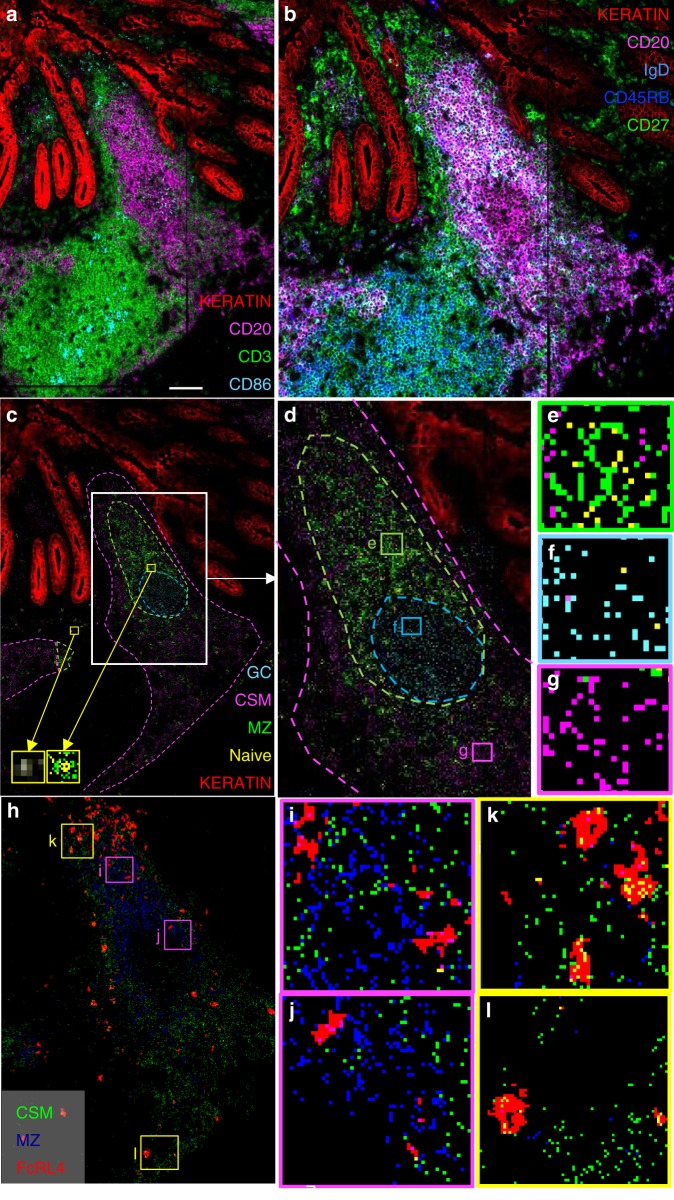
Marginal zone and class switched memory B cells occupy different microanatomical niches. **a** Visualisation of microanatomy of human appendix by imaging mass cytometry. A representative example of appendix from three different donors studied. CD3 (T cells; green), CD20 (B cells; magenta), CD86 (dendritic cells; cyan) and keratin (epithelium; red). Scale bar represents 100 μm. **b** Conventional mixing of fluorochromes did not enable the identification of B cell subsets that require multiple markers. The combination of CD27 (green), CD45RB (blue), CD20 (magenta) and IgD (cyan) would require the further addition of IgM for the detection of marginal zone B cells and their putative precursors. **c** Pixels with signal representing CD19^+^CD20^+^CD27^+^IgM^+^IgD^+^ of MZ B cells are green (approximate population boundary marked with a green dotted line). Pixels with signal representing CD19^+^CD20^+^CD27^+^IgM^−^IgD^−^ class switched memory (CSM) cells are magenta (approximate population boundary marked with a magenta dotted line). Importantly, pixels representing MZ and class switched memory have different distributions. Pixels with signal representing CD19^+^CD20^+^CD10^+^IgD^−^ of germinal centre (GC) cells are cyan (circled by a cyan dotted line). In addition, pixels representing CD19^+^CD20^+^CD27^−^IgM^+^IgD^+^ naïve B cells are yellow and pixels representing naïve cells are also illustrated as inserts. The epithelium is red. **d** An enlargement of the area of **c**. identified by the white rectangle. Pixel detail of the zones encircled by coloured dotted lines is illustrated in **e**–**g**. **h** Expression of FcRL4 in human appendix in red. Pixels with signal representing CD19^+^CD20^+^CD27^+^IgM^+^IgD^+^ of MZ B cells are blue. Expression of FcRL4 by MZ B cells is visualised as magenta pixels in **i** and **j**. Pixels with signal representing CD19^+^CD20^+^CD27^+^IgM^−^IgD^−^ class switched memory cells are green and so expression of FcRL4 by class switched memory cells is visualised as yellow pixels on FcRL4+ cells in **k** and **l**. FcRL4+ cells can therefore be either MZ or class switched memory cells

GALT zonal structure was visualised by cytokeratin (epithelium), CD3 (T cells), CD20 (B cells) and CD86 (dendritic cells) by imaging mass cytometry (Fig. 4a). The visualisation of B cell subsets by colour mixing generated complex patterns that were difficult to interpret even when only a selection of the required markers was used (Fig. 4b). We therefore designed an alternative strategy to identify the distribution of B cell subsets as single colour pixels on an image as summarised in Supplementary Fig. 5. We validated our subset classifications by comparing the expression of CD45RB and CD24 that were not used for B cell subset designation in image mass cytometry between mass cytometry and imaging mass cytometry, and found that these agreed (Supplementary Fig. 6).

The positions of pixels indicating CD27^+^IgM^+^IgD^+^ MZ B cells, CD27^+^IgM^−^ class switched memory cells (CSM), CD27^−^IgM^+^IgD^+^ naïve B cells and CD10^+^IgD^−^ GC cells are illustrated in Fig. 4c–g. The pixels representing CD27^+^IgM^−^ cells were located on the periphery of the lymphoid tissue (Fig. 4c, d, g), surrounding the CD27^+^IgM^+^IgD^+^ MZ population, demonstrating for the first time that these populations occupy different microanatomic niches (Fig. 4c–e). CD27^+^IgM^−^ cells also extended around the serosal aspect of the GC and around the T cell zone (Fig. 4c). CD27^+^IgM^+^IgD^+^ and some CD27^−^IgM^+^IgD^+^ naïve B cells occupied a space closest to the GC and the CD27^−^IgM^+^IgD^+^ naïve B cells also extended around the T cell zone. Sparsity of naïve B cells in the zone between the GC and the follicle-associated epithelium was observed in 2 of the 3 specimens of appendix analysed Supplementary Fig. 7, suggesting that some cells often considered to be naïve by observation of IgM and IgD co-expression in tissues can be misidentified without consideration of CD27.

High expression of FcRL4 and CD80 has previously been associated with activation and epithelial localisation, and their relatively high expression on CD27^+^IgM^+^IgD^+^ cells (Fig. 3d) suggested that these may be more closely associated with epithelia than CD27^+^IgM^−^ cells. However, this was not evident in tissue sections, where it was rather CD27^+^IgM^−^ cells that tended to occupy the sub-epithelial space (Fig. 4d). Visualisation CD27^+^IgM^+^IgD^+^ and CD27^+^IgM^−^ cells together with FcRL4 revealed that the FcRL4^+^ population included representatives of both subsets (Fig. 4h–l). CD80 and CD86 have been observed on B cells in the follicle-associated epithelium and sub-epithelial dome region of GALT previously^25^. Unfortunately, CD80 gave no signal in tissues and CD86 was not identified on B cells other than in the GC by imaging mass cytometry (Supplementary Fig. 8). The distributions of CD27^+^IgM^+^IgD^+^ cells and their CD27^−^CD45RB^+^ precursors were similar in tissue (Supplementary Fig. 9). The CD27^+^IgM^+^IgD^−^ (IgM only) population was distributed amongst both CD27^+^IgM^+^IgD^+^ and CD27^+^IgM^−^ populations (Supplementary Fig. 10). Intraepithelial B cells in follicle-associated epithelium had diverse phenotypes, demonstrating that this niche is not occupied by a single B cell subset (Supplementary Fig. 11)

### Human CD27^+^IgM^+^IgD^+^ B cells are widely disseminated

As discussed above, mass cytometry showed that CD27^+^IgM^+^IgD^+^ MZ cells are phenotypically similar to their CD27^−^CD45RB^+^ precursors but distant to memory cell subsets. In addition, imaging mass cytometry demonstrated that CD27^+^IgM^+^IgD^+^ cells have a different microanatomical distribution to CD27^+^IgM^−^ class switched memory cells. To further investigate the spatial distribution of CD27^+^IgM^+^IgD^+^ vis-á-vis memory B cell clones and how they relate to ongoing responses in GALT GC within and between GALT sites we used *IGHV* gene sequencing of sorted cells.

Three distant GALT sites and peripheral blood were sampled from four separate healthy individuals. The GALT sites spanned a metre of intestine, and included Peyer’s patches in the terminal ileum (PP), the lymphoid tissue around the periappendiceal orifice (A) and the boundary of the colon and rectum (CF) (Supplementary Fig. 12). Although separate precise sampling of these sites limited the cell numbers from each site available for analysis, it allowed observation of clone dissemination and clone sharing that could only occur via cellular recirculation through lymph and blood.

An initial flow cytometric analysis showed that GALT B cells from PP, A and CF had very similar phenotypes to each other and each was consistent with analysis of GALT by mass cytometry (Supplementary Fig. 13). Following the scheme in Supplementary Fig. 14 we used PCR with MID tagged second round primers to the constant region, to amplify Ig isotypes separately^28^. We analysed GC, CD27^+^IgD^+^ and CD27^+^IgD^−^ cells from each site of GALT and CD27^−^IgD^+^ naïve B cells, CD27^+^IgD^+^ and CD27^+^IgD^−^ cells and plasmablasts from blood. After quality control and initial bioinformatics analysis of IgH gene sequences the data comprised in total 80,710 unique sequences that were clustered into 50,862 different clonal families. Identical sequences were very rarely (0.006%) shared across multiple donors, consistent with the frequency of previously observed rare ‘public’ clones^29^ (Supplementary Fig. 15). With the exception of blood CD27^−^ naïve B cells, all subsets expressed mutated IgHV gene rearrangements with no obvious differences within a subset irrespective of tissue site^5,30^ (Supplementary Fig. 16). B cells from the CD27^+^IgM^+^IgD^+^ subset, whilst harbouring mutated IgHV, had a lower frequency of mutation than any of the CD27^+^IgD^−^ subsets, the GC cells or blood plasmablasts and IgM-only cells had slightly lower mutation frequencies than class switched cells, consistent with studies of these cells from blood (Supplementary Fig. 16). No obvious differences in IgHV family usage biases were observed (Supplementary Fig. 17).

**Fig. 5:**
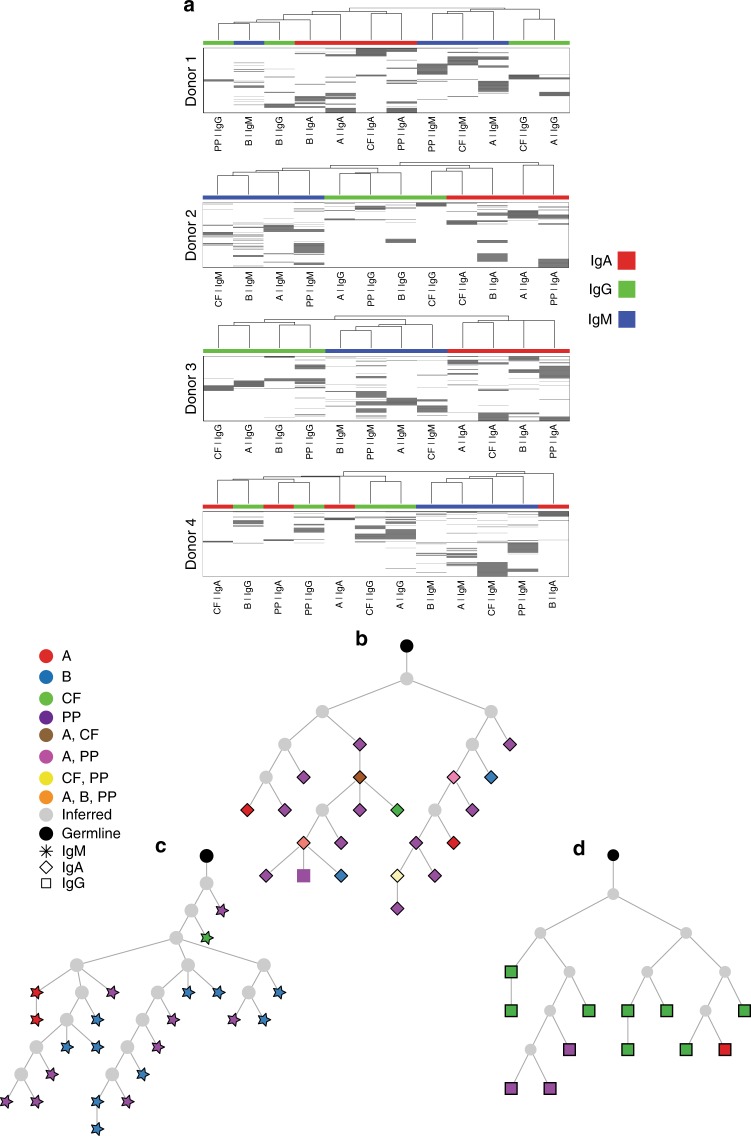
Dissemination of B cell clones through human gut. Analysis of B cells from three anatomical sites from each of four tissue donors. **a** Hierarchical clustering of isotype-specific B cell clones at different sites in the gut and blood. Each row represents a single clone, and columns represent site-isotype combinations. The grayscale represents the log_10_ transformed number of unique sequences in each clone. The hierarchical clustering is based on a distance function that quantifies that extent of shared clones across site-isotype combinations, and is shown on the top of the heatmap as a dendrogram. **b**–**d** Lineage trees showing dispersed clones of predominantly single isotype comprised of IgA (diamonds), IgM (stars) and IgG (squares). Recombining segments and junctional sequence of the most recent common ancestor (MRCA) of the clones are: **b** IGHV3-30, IGHJ4*02 TGTGCGAAAAAGGTTGSGGGAGGTCCGAAAACAGAAG GGGTTGACTACTGG. **c** IGHV3-23, IGHJ4*02 TGTGCGAAAGGTAGCGGGTCRKCTCGCCCGTACTACTTTGACTACTGG. **d** IGHV5-51*01, IGHJ3*02 TGTGCGAGACTTGACGGTAGCAGCAGTACTGATTGTCTTGATATYTGG

Identical sequences and clonally related sequences were observed between spatially separated sites within each donor (Supplementary Fig. 15). Disseminated B cell clones were more likely to be related to distant cells of the same isotype than to those of other isotypes present in the same microenvironment (Fig. 5a). Evidence of somatic diversification within an isotype was apparent from clonal lineage trees reconstructed based on shared somatic hypermutation patterns (Fig. 5b). This demonstrates migration of GALT CD27^+^ B cells expressing all isotypes studied through the gut and their somatic diversification after undergoing class switch recombination.

**Fig. 6:**
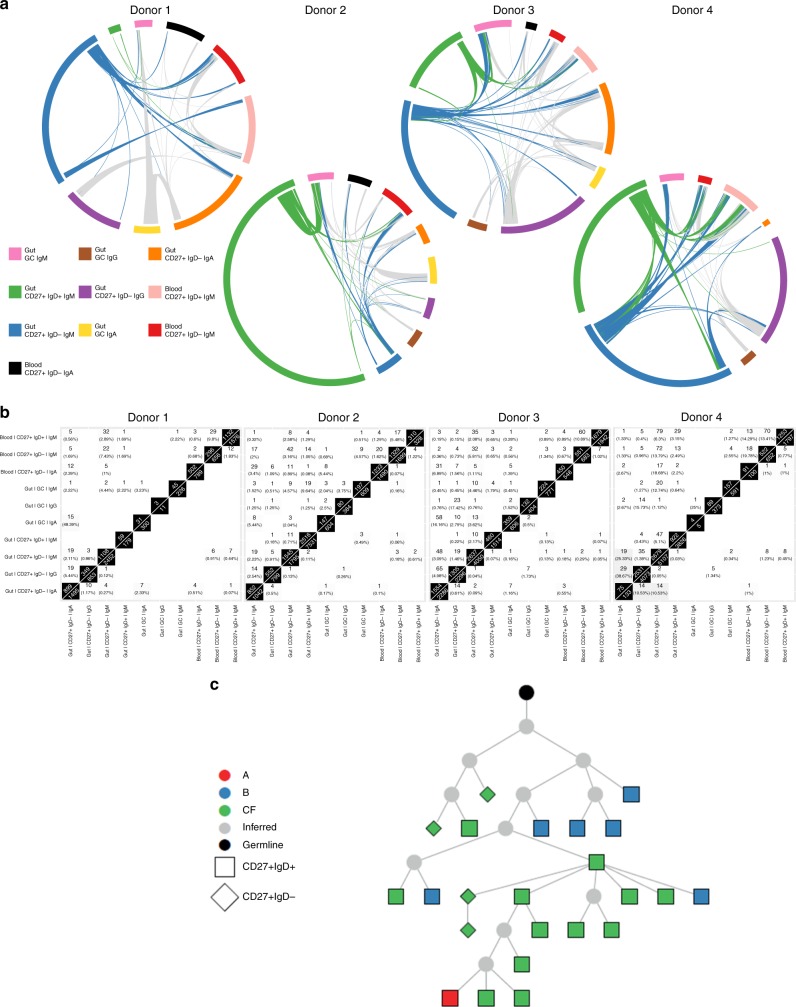
Clonal dissemination of B cell subsets. Analysis of B cells from three different sites from each of four tissue donors (**a**) Circos plots showing the clonal relationships between B cells in the gut and CD27^+^IgM^+^ subsets and CD27^+^IgA^+^ cells in blood for Donors 1–4. Sequences clonally related across tissue-isotype-subset are connected by lines, with sequences spanning gut CD27^+^IgM^+^IgD^+^ and gut CD27^+^IgM^+^IgD^−^ subsets coloured green and blue, respectively. All other connections are coloured light grey. **b** Numbers and percentages illustrating distribution of clone members (top triangles) and unique sequences (bottom triangles) from B cell subsets across different sites sampled. **c** Lineage tree depicting a shared lineage of blood and GALT CD27^+^IgM^+^IgD^+^ cells. Recombining segments of the clone were IGHV5-51*01, IGHJ4*02, junctional sequence TGTGCGAGACACGAGATGGAAGTGGCTGGTGCT TACCTTGGCTTCTGG

**Fig. 7:**
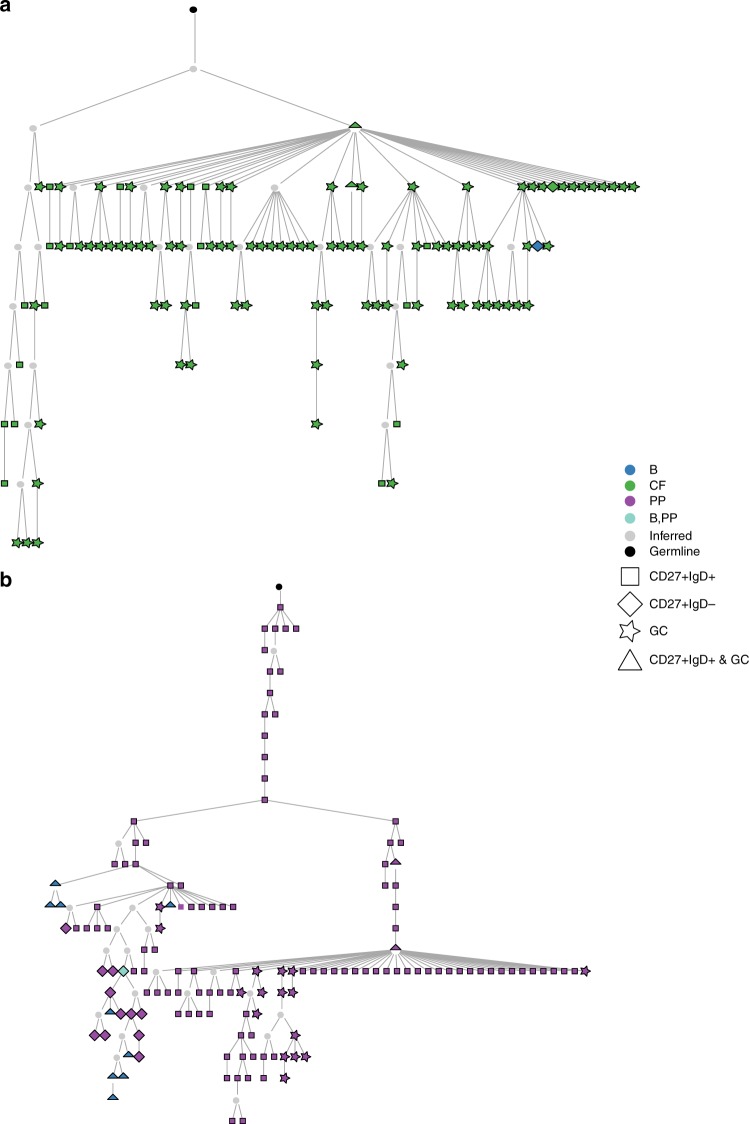
CD27^+^IgM^+^IgD^+^ cells diversify their receptors in gut GC. **a**, **b** Lineage trees including clone members isolated from GC of GALT, CD27^+^IgM^+^IgD^+^ cells from GALT and in (**b**) also CD27^+^IgM^+^IgD^+^ cells from blood. CD27^+^IgM^+^IgD^+^ cells before and after the GC phase were identified, indicating that the response is current and dynamic and generating cells enter and exit the GC response with the same (CD27^+^IgM^+^IgD^+^) phenotype. CD27^+^IgM^+^IgD^−^ clone members can also be identified. Recombining segments and junctional sequences of the MRCA of the clones are: **a**, IGHV3-33, IGHJ4*02 TGTGCGAGAGGGATCAA CTACGGTGACGCCGACGGCTTT GACAACTGG. **b**, IGHV5–10*01, IGHJ4*02 TGTGCGAGACATTTTGGGCACTACTTTGACTACTGG

In addition, clonal relationships between B cells from different subsets were apparent in all donors. GALT CD27^+^IgM^+^IgD^+^ and CD27^+^IgM^+^IgD^−^ B cells were both associated with clones that spanned GALT and blood. These subsets were also clonally related to each other within GALT (Fig. 6). Importantly, CD27^+^IgM^+^IgD^+^ and CD27^+^IgM^+^IgD^−^ clones both contained members that could be found within the GC, and lineage tree analysis identified ongoing diversification. CD27^+^IgM^+^IgD^+^ B cell clone members occurred before and after the GC stage in developmental terms and clone members were also identifiable in blood (Fig. 7). It has previously been suggested that CD27^+^IgM^+^IgD^+^ B cells can undergo somatic hypermutation outside GC. However, whereas AID expression by sorted GC cells was readily detected by quantitative PCR, neither CD27^+^IgM^+^IgD^+^ nor CD27^+^IgM^+^IgD^−^ GALT B cells expressed AID (Supplementary Fig. 18). Thus, these results support trafficking of GALT CD27^+^IgM^+^IgD^+^ and CD27^+^IgM^+^IgD^−^ B cells through blood and GALT including the GC microenvironment where they diversify their IGHV genes demonstrating a source of the enigmatic IgHV mutations that are characteristic for this population.

### Human MZ B cells diversify their IGHV genes in GALT

**Fig. 8:**
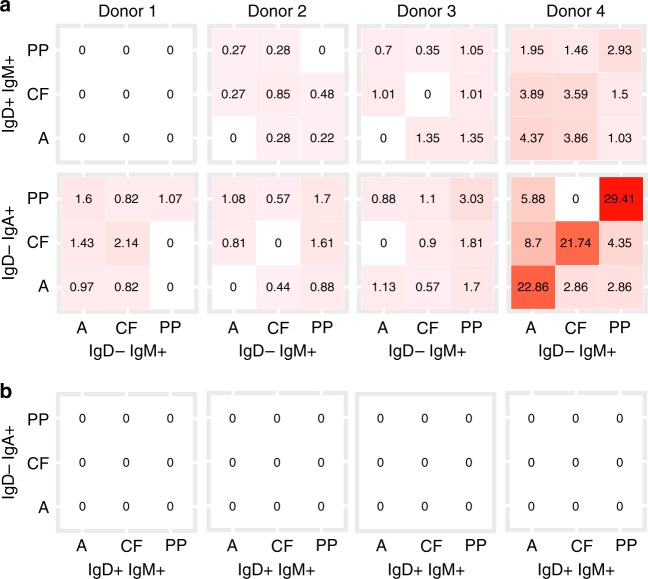
Analysis of B cells from three different sites from each of four tissue donors. Percentage of sequences with clonal relatives shared between CD27^+^IgM^+^IgD^+^ and CD27^+^IgM^+^IgD^−^, CD27^+^IgA^+^ and CD27^+^IgM^+^IgD^−^ subsets and CD27^+^IgA^+^ and CD27^+^IgM^+^IgD^+^ subsets, within and between sites. Numbers in blocks indicate the percentage of shared sequences within or between sites, with darker shading indicating increased sharing

While GALT CD27^+^IgM^+^IgD^+^ and CD27^+^IgM^+^IgD^−^ clones both contained members that could be found within the GC, their association with IgA expressing cells was clearly different. No GALT IgA^+^ members were identified among the 3957 GALT CD27^+^IgM^+^IgD^+^ containing clones (range 59–2516 clones) identified across all three sites in four donors. This was significantly different from GALT CD27^+^IgM^+^IgD^−^ containing clones where 105 of 7969 (range 19–48 of 1089–2660 clones) included GALT CD27^+^IgA^+^ members (combined *P* value < 10^−8^ by Fisher’s method) (Fig. 8). Of the 3957 clones that included CD27^+^IgM^+^IgD^+^ cells in GALT, 26 (0.7%) had clone members in GC, but none of these contained IgA^+^ clone members. Of 7969 clones including CD27^+^IgM^+^IgD^−^ cells in GALT, 59 (0.7%) had clone members in GC, of which 16 had GC IgA^+^ clone members. The frequency of IgA switched variants amongst the GC cells was significantly higher if clones contained CD27^+^IgM^+^IgD^−^ cells compared to clones containing CD27^+^IgM^+^IgD^+^ cells (combined *χ*^2^ *P* value = 0.0065 by Fisher’s method). While CD27^+^IgM^+^IgD^−^ cells were observed to be clonally related to both CD27^+^IgM^+^IgD^+^ cells and IgA variants, these relationships occurred in distinct sets of CD27^+^IgM^+^IgD^−^ clones.

## Discussion

Here we provide a multidimensional view of human B cells in lymphoid tissues that separates classical memory B cells from MZ B cells. For the first time, we observed a phenotypic progression from the naïve B cell pool via CD27^−^CD45RB^+^ MZ precursor cells to MZ B cells. This was evident in all tissues though we found no evidence for any site studied being specifically associated with progression through these stages. Although positioned closely to transitional cells in the viSNE plots, the MZ precursor cells did not form a phenotypic continuum with the transitional pool in the SPADE analysis, suggesting that they are not strictly T3 transitional cells that are formed as cells progress from T1 and T2 stages to become naïve cells. Rather, they appear to be later precursors in developmental terms that have acquired some properties of naïve B cells^8,9,31^. The developmental sequence is consistent with the observation of a relatively high abundance of CD27^−^CD45RB^+^ in children, in emerging B cells following hematopoietic stem cell transplantation and with differentiation of MZ B cells from splenic CD27^−^CD45RB^+^ precursors by NOTCH2 ligation^8,9^. It cannot be determined at this point whether NOTCH2 ligation is restricted to the spleen or can also occur in GALT, and if other signals in addition to NOTCH2 ligation can mediate the selection into the MZ lineage.

By imaging mass cytometry we observed that class switched memory B cells and MZ B cells are both present in GALT but that they have different microanatomic distributions. Whilst class switched memory cells were distributed around the periphery of the entire lymphoid tissue, MZ B cells had a more confined distribution between the GC and the follicle-associated epithelium. The area containing MZ B cells had class switched memory cells on all boundaries except at the interface between the MZ and the GC. The distribution of memory cells around the periphery of the tissue would place them on the antigenic front line in close proximity to potential pathogens. The significance of the distribution of MZ cells internal to the memory subset but adjacent to the GC is unclear, though resemblance between cells in this area and GC cells has been noted previously^25^. The associated expression of CD80/CD86 by B cells between the GC and follicle-associated epithelium in this study was confirmed here by mass cytometry while not by imaging mass cytometry.

As described before, we confirm that CD45RB was expressed by class switched CD27^+^ and CD27^−^ subsets of memory cells^17^. Hence, it is interesting to note that a population of CD27^−^IgM^+^IgD^−^ cells were identified here that were phenotypically similar to CD27^−^ and CD27^+^ subsets of IgA memory cells and that included cells that were CD45RB^+^. It is possible that this represents a population of CD27^−^IgM^+^ memory cells with highest relative abundance in the gut that is currently unappreciated and that require further attention.

Based on IGHV gene analysis, we observed that IgM-only cells could be clonally related to both MZ and memory B cells but not within the same clone in GALT. Moreover, by imaging mass cytometry we observed that IgM-only cells shared both MZ and class switched memory microanatomical biases. Taken together, this suggests that IgM-only cells in GALT can belong to either the MZ or memory developmental axis. This is in contrast to Bagnara et al. who proposed that IgM memory B cells can be either CD27^+^IgM^+^IgD^−^ or CD27^+^ IgM^+^IgD^+^ in blood and spleen^5^. We show that in terms of derivation, microanatomy and clonal segregation, CD27^+^IgM^+^IgD^+^ are not synonymous with classical memory cells but that IgM-only cells can belong to either compartment and cannot currently be assigned to either compartment by phenotype alone.

The IGHV gene analysis also demonstrated that GALT MZ and class switched memory B cells were rarely clonally related which is consistent with our observations made by mass cytometry and imaging mass cytometry, highlighting that the two populations represent separate developmental pathways. We cannot exclude the possibility that the total lack of IgA variants within CD27^+^IgM^+^IgD^+^ clones was due to the limited number of cells harvested from each site from each individual. However, the observation that similar frequencies of CD27^+^IgM^+^IgD^+^ and CD27^+^IgM^+^IgD^−^ clones were associated with the GC argues for our ability to detect clonal relationships between these small, accurately sampled populations. Thus, even though we cannot exclude switching, our data demonstrate that if such switching occur, it is less prevalent among GALT MZ lineage cells than B cells differentiating along a conventional memory developmental axis.

Circulating and splenic CD27^+^IgM^+^IgD^+^ B cells have mutations in their IGHV genes implying previous GC exposure^5,32,33^. This contrasts with mouse MZ B cells that differentiate from T2 cells in spleen and do not have this feature^34^. Based on the presence of CD27^+^IgM^+^IgD^+^ cells with mutations in their IGHV genes in CD40L deficient patients, it has been suggested that mutations in IGHV genes in CD27^+^IgM^+^IgD^+^ cells are GC and potentially even AID independent^1,35^. Our study does not exclude these possibilities. However, we show that in healthy individuals, mutations can be introduced into MZ B cells in GALT GC. We detected CD27^+^IgM^+^IgD^+^ B cells that were clonally related to GC B cells, and we were able to detect AID in isolated GC cells from GALT but not CD27^+^IgM^+^IgD^+^ B cells. GC are present in GALT from the first days after birth and the small abortive GC that can occur in CD40L deficient patients could potentially support some mutation of IGV genes in GALT^36,37^. Overall, our results support a model where mutations in IGHV genes of CD27^+^IgM^+^IgD^+^ B cells are acquired in the GCs of GALT. In this case, mutations in *BCL6* in CD27^+^IgM^+^IgD^+^ MZ cells observed by others would also be acquired in GC of GALT^6^, and for the reasons stated above mutated BCL6 in CD27^+^IgM^+^IgD^+^ B cells does not warrant their classification as classical memory B cells.

Consistent with other work, we show that class switched memory B cells are the most abundant B cells in human GALT and that most express IgA^15^. By IGHV gene analysis we show that clones of memory B cells disseminate through the gut and blood, spanning distant sites over one metre of intestine, consistent with distribution of mucosal memory B cells in mice. These clones continue to expand and evolve in the GC but we observe no overarching tendency for local accumulations other than in GC. This distribution of memory cells occurs despite the vastly different antigenic environments from which cells are isolated and the different bacterial species the lymphoid tissue will be exposed to in the ileum and the colon. This suggests that classical gut memory cells arising from exposure to infection or vaccine could disseminate to provide wide coverage and rapid immune protection over time, consistent with intestinal memory B cell persistence in mice^38,39^.

It is interesting to note that B cells that would be classified as the same subset according to expression of conventional groups of markers can differ in their expression of other functionally relevant antigens depending on location. Splenic B cells tended to express higher levels of CD21 and CD180, both associated with innate B cell responses, compared to equivalent subsets in the tonsil or GALT. This implies that such antigens can be locally enhanced as B cells enter tissues or that separate migratory subsets of cells exist that otherwise appear equivalent. This will be an important area for future investigation.

In summary, by spatiotemporal analysis of B cells in tissues we visualise phenotypic progression from CD27^−^CD45RB^+^ precursors to marginal zone B cells in humans separate from memory cells, and that marginal zone and memory B cells occupy different microenvironments in GALT. Human MZ B cell development involves a stage of clonal expansion and repertoire diversification in the GC of GALT, thus endowing them with signatures of GC experience that were previously unexplained.

## Methods

### Tissue samples

All tissues used in this study were collected with ethical approval from UK Research Ethics Committees administered through the Integrated Research Application System. All samples were collected with informed consent. Gut biopsies (PP, A, CF) and paired blood samples were from 45 healthy patients undergoing investigative colonoscopy who had no evidence of inflammation or other pathology. Details of age of these tissue donors is in Supplementary Fig. 12b. In addition, non-inflamed tonsils from five adult patients were collected from surgical theatres. Normal spleen samples were from six cadaver organ donors. The three snap frozen samples of normal appendix used were from two patients with cancer who had undergone surgical right hemicolectomy (appendix 1 and 3) and one cadaver donor (appendix 2). Surgical samples of spleen, tonsil and appendix were all anonymous.

Mononuclear cells from gut biopsies and paired peripheral blood and from tonsil and spleen were isolated as previously described^40^. Tonsil and spleen cell suspensions were cryopreserved before use.

### Mass cytometry

Antibodies to be used for mass cytometry (Supplementary Table 1) were either purchased ready tagged from Fluidigm or else purchased protein free from the suppliers as indicated in Supplementary Table 1 and conjugated in house using MaxPar labelling kits from Fluidigm according to the manufacturer’s instructions. For viability staining, 2 × 10^6^ cells were washed in PBS containing 0.5% BSA with 2 mM EDTA (cell-staining medium: C-SM) and incubated for 20 min in 1 ml rhodium intercalator (Fluidigm) diluted 1:500 in PBS. Cells were washed twice in C-SM, prior to resuspending the cell pellet in 10 mcl Kiovig Fc block (5 mg/ml; Baxter) for 10 min at room temperature followed by staining with the panel of metal-conjugated Abs using previously titrated concentrations, in a total volume of 100 μl on ice for 30 min. After two further washes in C-SM cells were fixed in 2% PFA in PBS at 4 °C overnight. Cells were centrifuged and counterstained with 500 μl perm buffer (0.3% saponin (Sigma) in C-SM) and 100 μl intercalatin Ir (Fluidigm) for 30 min at room temperature. Cells were then washed twice with 1 ml PBS and twice with 1 ml Milli-Q water and resuspended in Milli-Q water with EQ beads (DVS Sciences) and the cell concentration adjusted to 0.5 × 10^6^ cells/ml. Data were acquired immediately using a CyTOF mass cytometer as described previously and files exported in flow cytometry file (FCS) format for analysis of data to Cytobank (https://mrc.cytobank.org).The effect of mild collagenase digestion on binding of antibodies by this method revealed that TACI, CD62L and CCR6 were reduced by digestion (Supplementary Fig. 19). Since cryopreservation was used for tonsil and spleen but not GALT or PBMC, the effect of cryopreservation on the method and of potential relative loss of populations such as plasmablasts was tested and found to have no apparent effect (Supplementary Fig. 20).

### Analysis of mass cytometry

FCS files were normalised using Nolan Lab Normalizer software (v0.3, available online at https://github.com/nolanlab/bead-normalization/releases). Pre- and post- normalisation plots are shown in Supplementary Fig. 1. FCs files were concatenated using The Cytobank FCS file concatenation tool (available online at https://support.cytobank.org/hc/en-us/articles/206336147-FCS-file-concatenation-tool). Concatenated normalised files were then uploaded onto the Cytobank (https://mrc.cytobank.org/) and gated as shown (Supplementary Fig. 1). A total of 144,367 live single CD19^+^ cells from GALT, spleen and tonsil (total 433,101) were then included in the viSNE analysis using CD10, CD24, CD27, CD38, IgA, IgD, IgM, IgG and HLADR. Manually gating the viSNE plots was difficult due to the gradient of expression of many markers. In addition, SPADE as a clustering tool alone was found to be poorly reproducible (Supplementary Fig. 21). Methods such as PhenoGraph^41^ (for example) generated interesting B cell subset data but this was difficult to tie to existing literature and other datasets and were therefore not pursued for this study (Supplementary Fig. 22). SPADE was therefore run on viSNE using tSNE 1 and tSNE 2 as clustering parameters allowing identification of B cell subsets by the arrangement of phenotypically similar nodes into SPADE bubbles.

Multidimensional scaling was used to visualise the phenotypic similarities and differences in the SPADE bubbles exported as .fcs files between biological samples and anatomical sites prior to concatenation^16^. Each point represents a sample, and the distances between the points are proportional to the Euclidean distance between the samples. Kruskal stress shows how much information was lost during the dimension reduction steps.

To test whether the CD45RB^+^ cells constitute a population with a distinctive phenotypic profile within the naive B cell population, a bootstrapping procedure (random sampling with replacement) was implemented. A number of cells equal to the number of cells observed in the CD45RB^+^ bubble for each tissue was randomly sampled from the naïve B cell SPADE bubble for 10,000 times. Each time, the median expression for each marker across all sampled cells was measured to derive the expected distribution of expression markers. This distribution was compared with the median expression values in the observed CD45RB^+^ population, and *z-*scores were used to determine if there is a significant difference between the two populations^18–20^.

### Imaging mass cytometry

Frozen sections of 4 μm thickness were cut onto glass slides and stored at −80 °C. Immediately before use they were raised slowly to 4 °C and fixed for 30 min in 4% PFA in PBS at 4 °C. Sections were washed twice in 37 °C 0.1% Tween-20 in PBS for 8 min followed by incubation in blocking solution (0.1% Tween-20, 1/20 FcR block [Trustain, Biolegend], 5 mg/ml human IgG [5% KIOVIG, Baxalta UK Ltd.] and 5% BSA in Superblock [Thermo Fisher Scientific]) for 1 h at room temperature. Sections were then stained overnight at 4 °C in pre-titrated primary antibodies diluted in blocking solution. Sections were then washed twice with PBS 0.1% Tween-20 for 8 min prior to counterstaining with 0.5 µM Intercalator-Ir (Fluidigm) diluted in PBS, for 30 min at room temperature. Following washes in PBS and water, slides were air dried prior to ablation. Data were acquired on a Hyperion imaging system coupled to a Helios Mass Cytometer (Fludigm), at a laser frequency of 200 Hz and laser power of 6 dB.

When the individual ablation regions were smaller than the region of interest, images were stitched together in MATLAB by shifting the *x* and *y* co-ordinates of sequential ablations to create a full region of interest. Initial analysis used MCD viewer to identify areas for further investigation by creating a number of overlaid images depicting a set of relevant markers. The inbuilt thresholding tools were used to clean up pixels with very low intensity values resulting from, for example, cross channel and system noise. Files were exported in TIFF format. These were then used to create Fig. 4a, b; Supplementary Fig. 7 and 11 in Fiji software (https://fiji.sc/).

The protocol for creation of Fig. 4c–l and Supplementary Fig. 8–10 is illustrated in Supplementary Fig. 5. Images created by imaging mass cytometry were stitched together when required in MATLAB and imported to MCD viewer. Data were exported as a stacked TIFF file from MCD viewer and converted to FCS files in MATLAB with each event representing a pixel and not a cell as in standard cytometry. FCS files were uploaded onto the Cytobank server. In Cytobank the *x* and *y* parameters were displayed on a linear scale and gates of the regions of interest were created. viSNE analysis was run on the pixels in these gates with all panel markers. SPADE was run on viSNE to identify nodes containing CD19^+^ CD20^+^ pixels. Nodes were grouped in SPADE bubbles and exported for further analysis. A sequential viSNE analysis was run using B cell markers CD10, CD19, CD20, CD24, CD27 CD38, CD45RB, IgM and IgD from the panel. SPADE was run on the second viSNE to group nodes into SPADE bubbles that corresponded to B cell subsets. The SPADE bubble named ‘other’ contained nodes that could not be placed confidently within SPADE bubbles representing known B cells subsets and were therefore excluded. Events within SPADE bubbles representing B cell subsets were exported from Cytobank and converted in MATLAB to a MCD Viewer readable text format. The B cell subset pixels were then mapped back to the region of interest and pseudocoloured in MCD viewer.

### Flow cytometry and cell sorting

All flow cytometry used appropriate isotype controls. Gating and compensation were aided by the performing fluorescence minus one controls. Cells isolated from biopsies or blood were stained with blue-fluorescent reactive dye (Life Technologies), CD19-BV785, CD27-APC (BD Biosciences) or PE or BV421, CD10-BV605 or APC, CD38-PerCp-ef710 (eBioscience), CD24-PE/Cy7 or BV605, IgD-APC/Cy7, IgM-V450 (BD Biosciences), IgG-PE/Cy7 and IgA-FITC (Miltenyi Biotec), in 2% FCS staining buffer with 1 mM EDTA for 15 min at 4 °C before analysis on the BD LSRFortessa (BD Biosciences). For high-throughput sequencing analysis, cells were stained with LIVE/DEAD Fixable Aqua (Life Technologies) or DAPI, CD19-BV785, CD27-FITC or APC, IgD-APC/Cy7, CD38-PerCp-ef710 (eBiosciences), CD10-BV605 in 2% FCS staining buffer with 1 mM EDTA for 15 min at 4 °C before sorted on the BD FACSAria (BD Biosciences). Four subsets from PBMC and three subsets from paired biopsy mononuclear cells from Donors 1 to 4. The numbers of cells used to generate sequences for each sample from Donors 1 to 4 are shown in Supplementary Fig. 14d.

### High throughput sequencing of immunoglobulin genes

RNA from sorted cells was isolated using an RNeasy Mini Kit (Qiagen) according to manufacturer’s instructions. For cells from Donors 1 to 4, cDNA synthesis was repeated eight times for each sample using SuperScript III First-Strand Synthesis Supermix (Invitrogen) according to manufacturer’s instructions (Supplementary Fig. 14c). The reaction was performed at 25 °C for 10 mins, 50 °C for 30 min, 85 °C for 5 min and then chilled on ice before adding RNase and incubation at 37 °C for 20 min. Immunoglobulin genes were amplified by nested PCR using isotype-specific primers as described previously: for VH1-6/7 leader (1st PCR; PCR1) and FW1-VH1-6/7 (2nd PCR; PCR2)^28,42^ in conjunction with nested, constant region primers. In brief, PCR1 was performed in 20 μl reaction mix containing 2 μl of cDNA, 10 μl of Q5 High-Fidelity 2X Master Mix (NEB), IGHV1-6/7 forward primers (83.2 nM each) and reverse primers (500 nM; either CHA″, CHG″ or CHM″ for IgA, IgG and IgM respectively). The nested PCR2 was then performed in 50 μl reaction mix containing 5 μl PCR1 products, 25 μl of Q5 High-Fidelity 2X Master Mix (NEB), forward FW1-VH1-6/7 primers (83.2 nM each) and nested reverse primers (500 nM), both tailed with 10-nucleotide multiplex-identifier (MID). Two rounds of PCR were performed with the same programme: 98 °C for 30 s, followed by 20 cycles of 98 °C (5 s), 60 °C (10 s), 72 °C (20 s) and final extension at 72 °C for 2 min. Primer sequences^28^ are in Supplementary Table 3.

After amplification, PCR primers were removed from the pooled (×8) products of each sample by electrophoresis and QIAquick Gel Purification Kit (Qiagen). The concentration of each sample was measured using Qubit fluorometer (Invitrogen). Samples to be pooled for sequencing were mixed in equal quantities, and further purified and concentrated using Silica Bead DNA Gel Extraction Kits (Thermo Fisher Scientific). Samples (including all isotypes from all sorted cell subsets) from the same gut biopsy site (e.g. PP) of four individual donors were pooled into one sequencing lane. Samples (including all isotypes from sorted CD19^+^CD27^+^ and plasmablast cell subsets) from PBMC of four individual donors were pooled as one for sequencing on one sequencing lane. Samples from CD19^+^CD27^−^sorted PBMC were separately pooled for another sequencing lane. Pyrosequencing was performed on the Roche 454 GS FLX + System by LGC Genomics (Germany). The procedure of sample preparation is illustrated in Supplementary Fig. 14c.

### Sequence analysis: processing raw reads

Bioinformatics analysis was carried out using the Immcantation framework (http://immcantation.readthedocs.io). Raw high-throughput sequencing reads were processed and filtered for quality using pRESTO^43^. Reads of length less than 250 and mean Phred quality score less than 20 were removed. Next, MIDs on the 5′ and 3′ were identified and annotated. Reads without exact sequence matches to the MIDs were removed. The forward (V region) and reverse (constant region) primers were matched with maximum error rate of up to 0.2. The forward primer regions were masked with Ns while the reverse primer regions were trimmed from the reads. Reads with identical nucleotide sequence, MIDs, forward and reverse primers were collapsed into a single sequence.

### V(D)J germline assignment and further quality control

Following processing of raw sequencing data using pRESTO Version 0.5.0-2015.07.29, germline V(D)J segments were identified using IMGT/HighV-QUEST Version 3.3.2^44^. Further quality control and advanced sequence analysis was carried out using Change-O^45^ and R (R Core Team, 2015). Sequences identified as non-functional by IMGT/HighV-QUEST and those with non-matching MIDs (suggesting they were chimeras) were removed. Finally, sequences with more than five mutations in a sliding window of 10 base pairs that were likely to be chimeric sequence artefacts were also removed (Supplementary Fig. 23a).

### Clonal grouping and lineage tree construction

Each set of sequences was partitioned into clonal groups using a two-step approach. Initially sequences were grouped based on common germline IGHV and IGHJ gene assignment and identical junction length. Within these larger groups, sequences differing from one another by a distance of less than 0.2 were defined as clones. Distance between sequences was measured as the number of nucleotide differences in the CDR3, with each difference weighted by the human S5F model of somatic hypermutation targeting and substitution^46^ as previously described^47^. This distance was normalised by dividing by the CDR3 length. The threshold of 0.2 was chosen by manual inspection of the distance-to-nearest plots generated for each individual (Supplementary Fig. 23b). Lineages were constructed for each clonal group containing at least two sequences as described^47^.

For formulation of heatmaps, the distribution of clones was calculated by dividing the number of sequences in each clone by the total number of sequences at the site. Hierarchical clustering was performed on the manhattan distance between distributions of clones using the R function hclust with Ward.D agglomeration method. The circos plots were created using the circlize package 0.3.10 in R^48^.

### Quantitative RT-PCR

Quantitative RT-PCR was as described previously TaqMan qRT-PCR assays (Applied Biosystems) consisting of amplification primers and fluorescently labelled MGB-probes were used to quantify AID expression^49^. qRTPCR was performed in triplicate for each sample with each gene multiplexed with an18s endogenous control (taqman assay labelled with different dye). Samples were run on a viia7 qPCR machine (Applied Biosystems) and subjected to ∆CT analysis relative to 18S expression.

### Code availability

All code used in the manuscript is available on request.

## Electronic supplementary material

Supplementary Information

## Data Availability

NGS data are available from NCBI under accession code: SRP161123; BioProject ID: PRJNA478215. Other data are available on request.
