# Supplementary material for: Spatiotemporal segregation of human marginal zone and memory B cell populations in lymphoid tissue
Source: Nat Commun. 2018 Sep 21;9:3857. doi: 10.1038/s41467-018-06089-1 (PMC6155012; doi:10.1038/s41467-018-06089-1)
Supplement: Supplementary file 1 — Supplementary Information [file 41467_2018_6089_MOESM1_ESM.pdf]

Spatiotemporal segregation of human marginal zone and memory B cell populations in lymphoid tissue

Zhao et al

Supplementary Information

Supplementary Figure 1

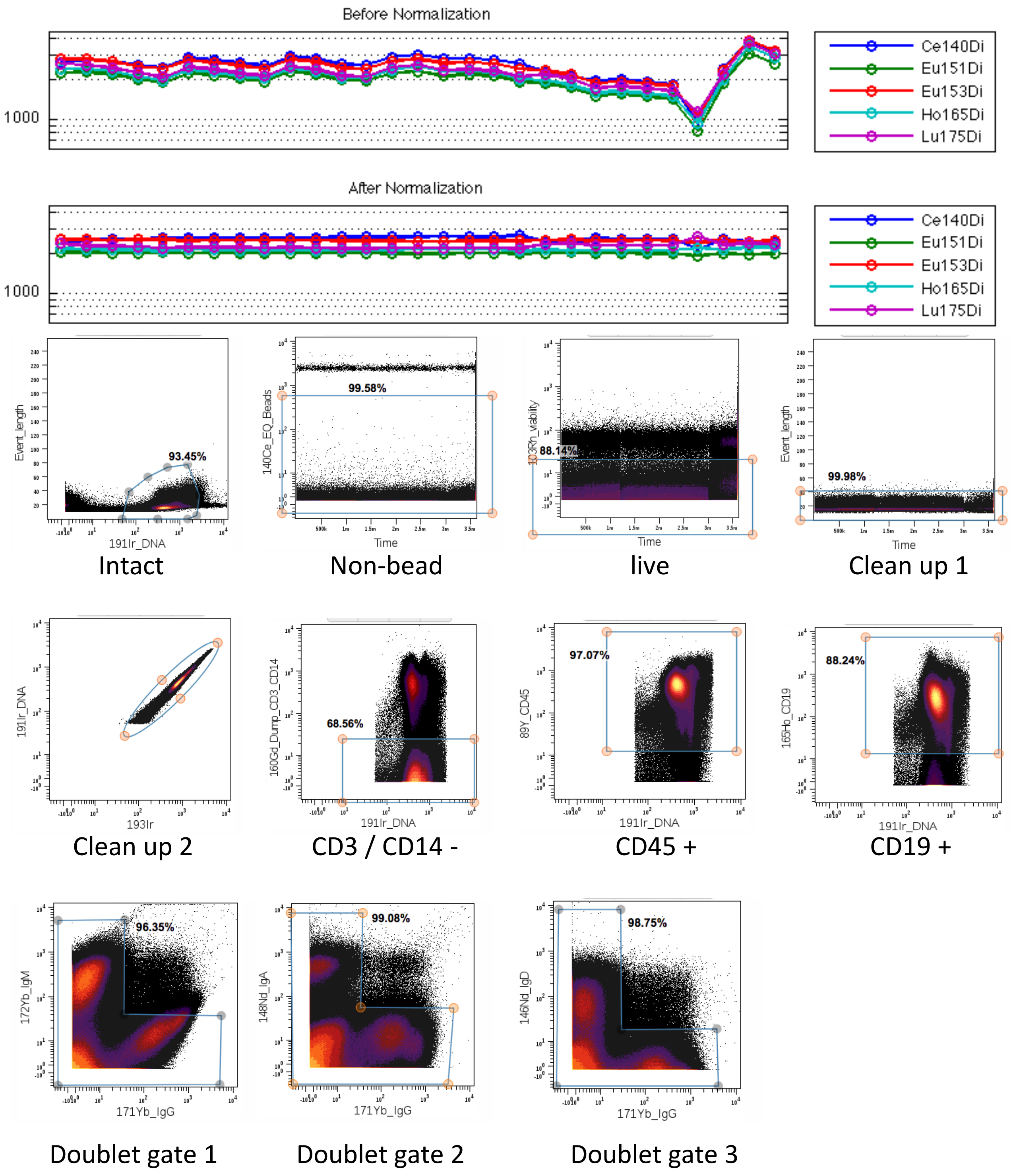

Supplementary Figure 1: Quality control and preliminary gating for mass cytometry. Bead normalization, doublet exclusion and preliminary gating for analysis of B cells isolated from tissues. A gate was created around intact cells that ensured that all relevant events in all tissues were captured. ‘Dump’ channels and selection of CD19+ cells excluded all but B cells from our analysis. Subsequent doublet gates 1,2 and 3 cells excluded the remaining minority of cells with implausible immunoglobulin combinations.

Supplementary Figure 2

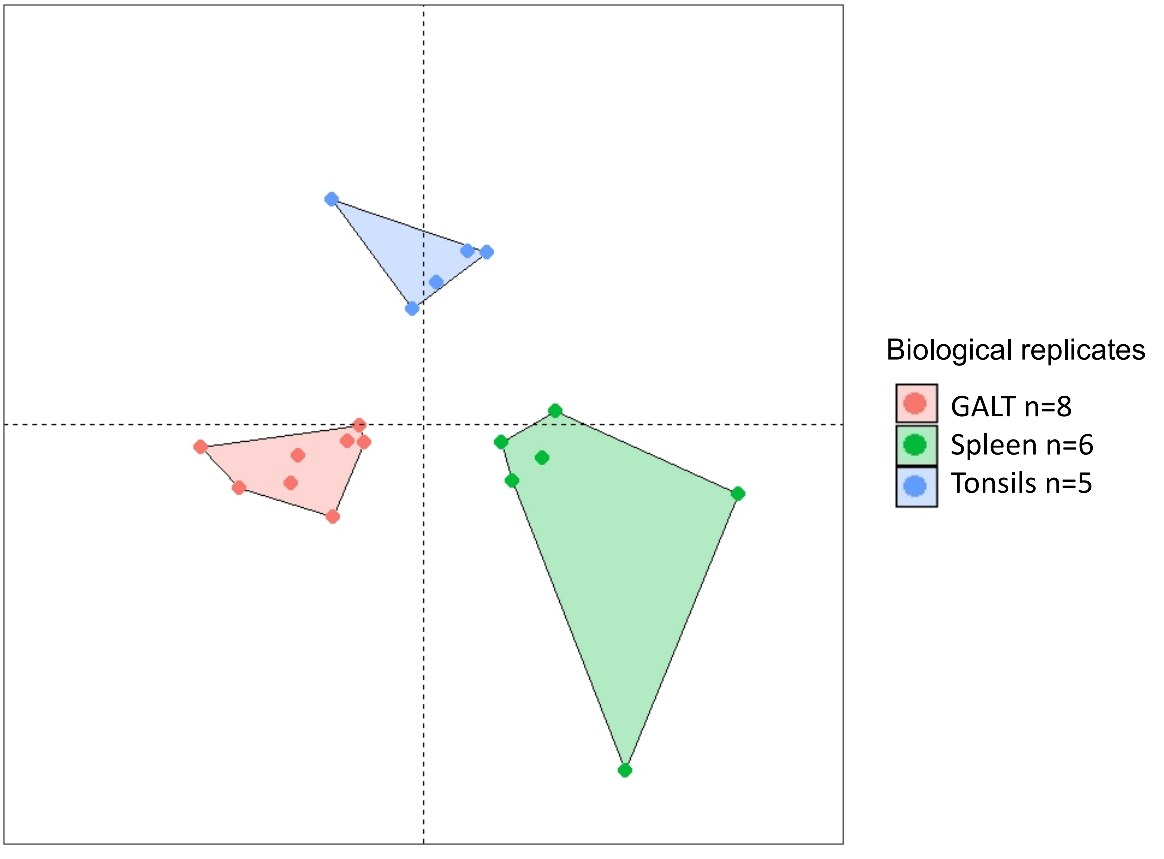

Supplementary Figure 2: Multidimensional scaling to compare tissue isolates.
Multidimensional scaling (MDS) representations to visualize the similarities between samples, based on their phenotype profile. Each dot corresponds to a biological sample and the distances between the dots are proportional to the Euclidean distances calculated based on the cell phenotype profiles. Kruskal stress is 8.56. Biological replicates of each tissue are clustered together.

Supplementary Figure 3

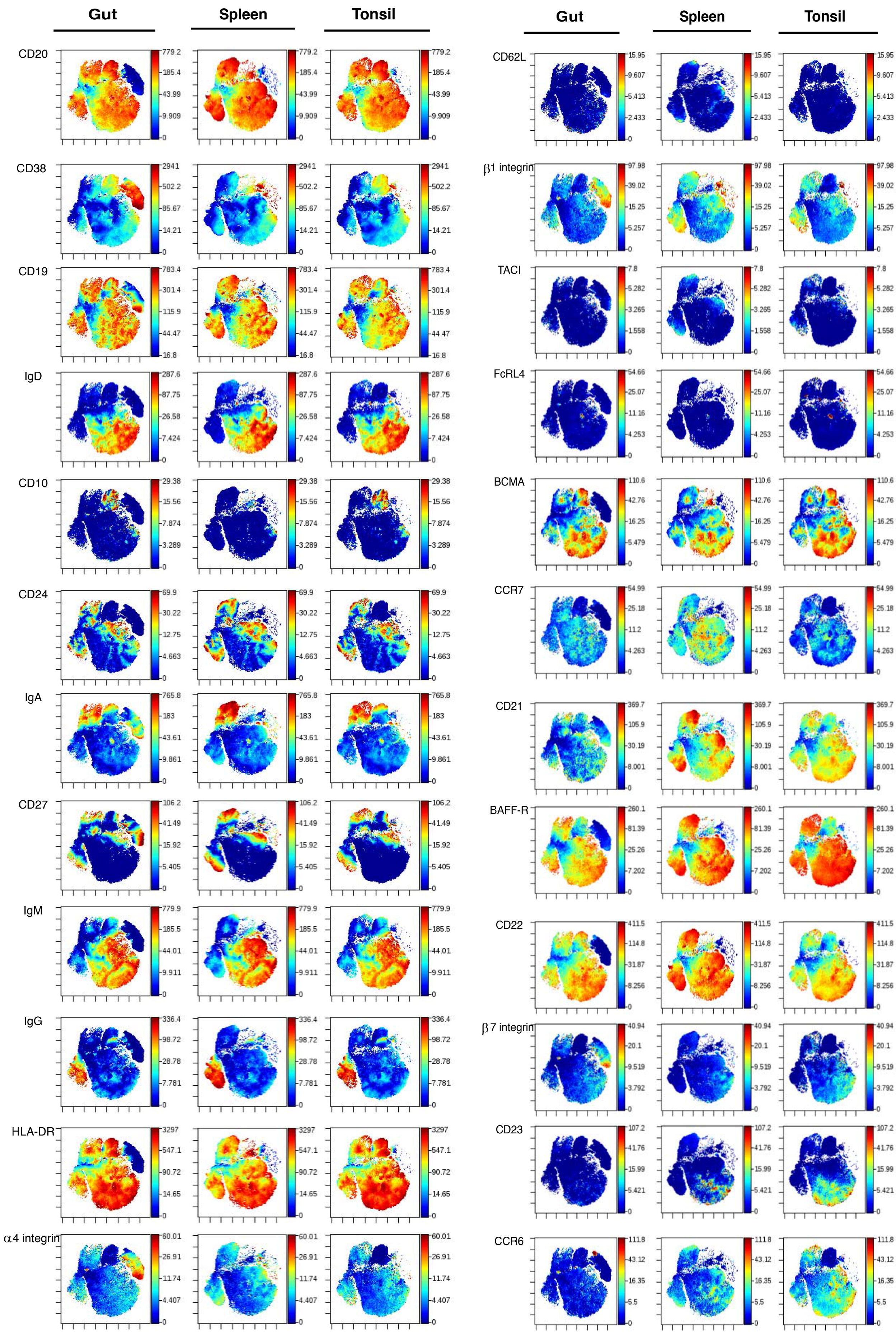

Supplementary Figure 3 (continued)

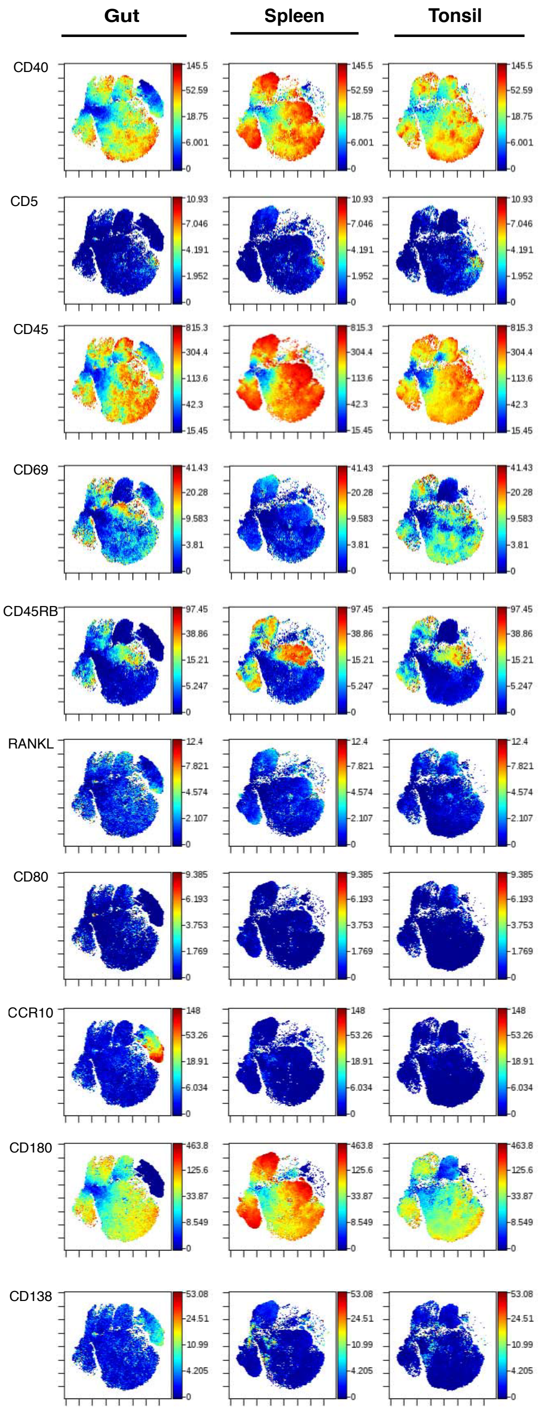

Supplementary Figure 3: Visualisation of marker expression in tissues by viSNE.

viSNE projections of B cells in tissues according to expression of CD27, CD24, CD28, CD10, IgM, IgD, IgA, IgG and HLA-DR for identification of B cell subsets. Expression of all markers analysed is visualised individually for each tissue.

Supplementary Figure 4

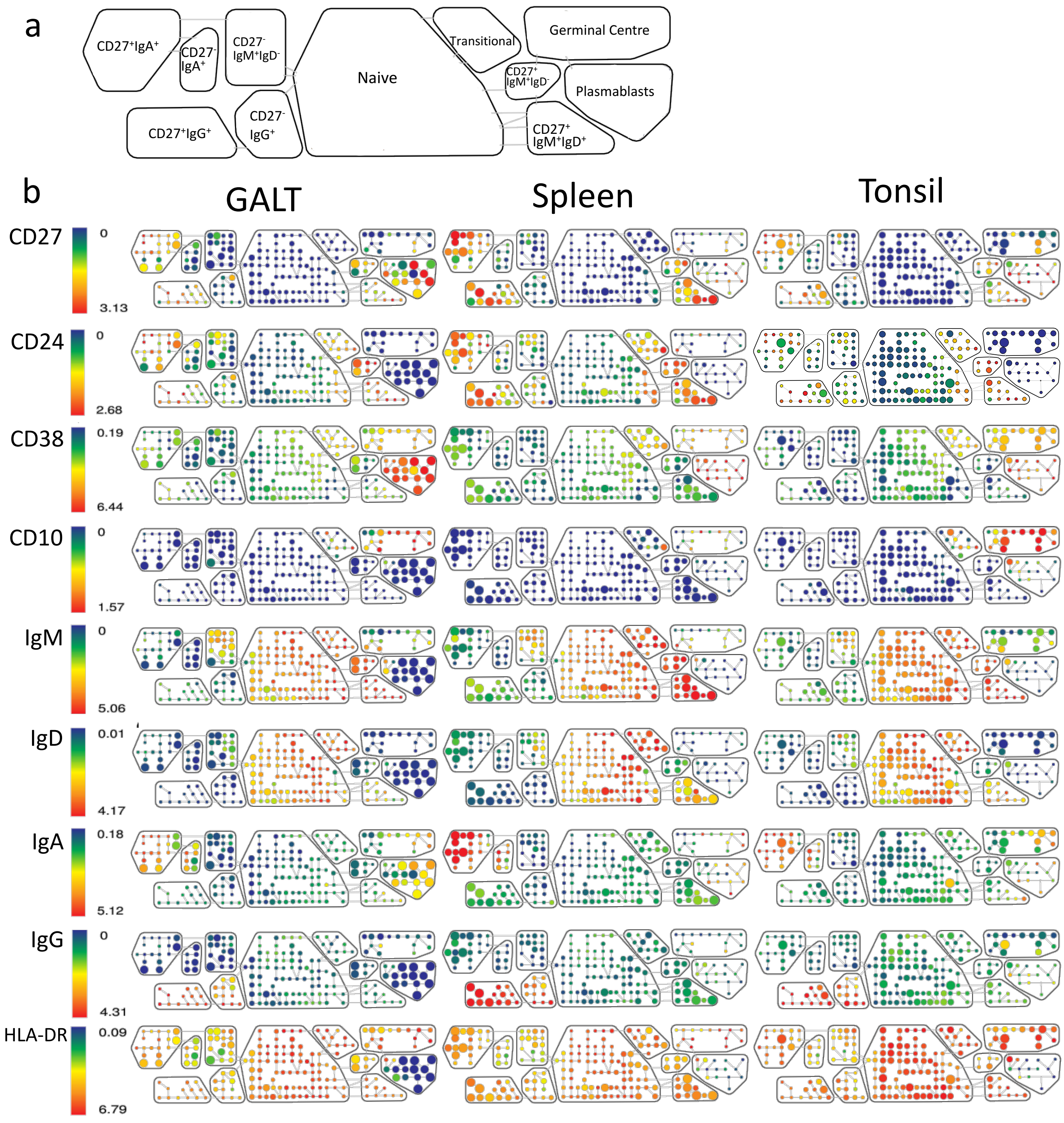

Supplementary Figure 4: SPADE plots illustrating the expression of markers used for clustering.

a. Key to B cells subsets identified in by SPADE plots in b. that were created for each tissue to enable comparisons of their compositions.

Supplementary Figure 5

Multiple imaging mass cytometry (IMC) images were obtained from the serial ablation of stained tissue sections, each generating approximately 1 million pixels with 1 pixel representing 1µM^2 of ablated tissue. Where regions of interest spanned individual ablations, these were stitched together in MATLAB before being visualized MCD viewer.

MCD viewer was used to visualize single or dual marker expression and merged using Fiji software (eg Fig. 4a and 4b; Supplementary Figs. 7 and 11). However, B cell subsets that are defined by the expression of multiple markers could not be visualized in this manner due to the complexity of colour mixing (illustrated in Fig. 4b), and the density of cells in lymphoid tissues precluded accurate cell segmentation. Therefore a new method was created that permitted representation of each B cell subset as a single colour by assigning individual pixels a phenotype defined by the median intensity of lineage markers as described below.

To enable this, data were exported as a stacked TIFF file from MCD viewer and converted to FCS files in MATLAB and uploaded onto the Cytobank server. This enabled individual pixels (rather than cells) to be visualized as single FCS events.

In Cytobank the x and y coordinates were displayed on a linear scale and gates of the regions of interest were created. Most regions were occupied by black space or non-B cells. In order to identify pixels representing B cells, a viSNE analysis using all panel markers was run. The example viSNE plot to the right demonstrates that the majority of the pixels did not express CD20 and thus did not represent B cells, and that due to the multiple areas of positive signal, direct gating in the viSNE was not possible.

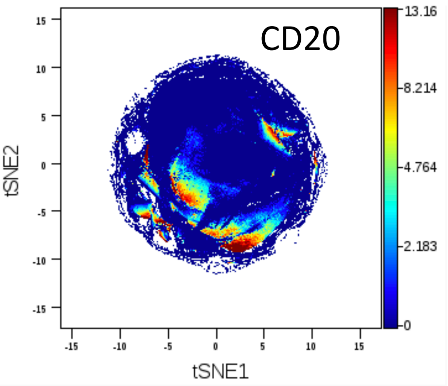

SPADE was therefore run on viSNE in order to manually select nodes containing CD19 and CD20 positive pixels. Pixels were assigned into a SPADE bubble and exported for the next stage of the analysis.

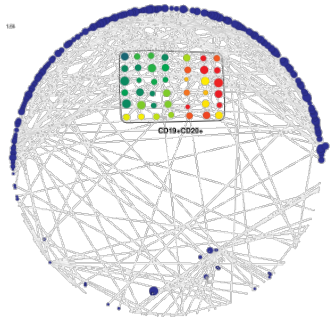

Continued…

Supplementary Figure 5 continued

A second viSNE analysis was run on the selected B cell pixels using B cell markers CD10, CD19, CD20, CD24, CD27 CD38, CD45RB, IgM and IgD. Again, owing to multiple foci of some lineage markers alongside the homogenous expression of others, it was not possible to directly gate B cell subsets on visne plots. SPADE run on viSNE was found to be the most accurate and reproducible method of depicting the data.

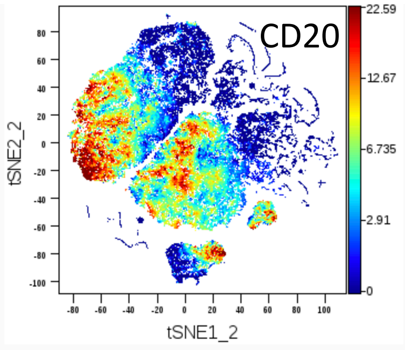

SPADE was run on the viSNE of CD19+CD20+ pixels and nodes containing pixels corresponding to B cell subsets were grouped into bubbles. The SPADE bubble named 'unassigned' contained nodes that could not be placed confidently within SPADE bubbles representing known B cell subsets and were therefore excluded. The numbers in the table below are the calculated raw values of medians for each marker within each subset bubble.

	CD27	IgD	IgM	CD45RB	CD10
CD27+IgM+IgD+	4.83	31.58	11.45	5.24	0.0
CD27+IgM+IgD-	6.26	0.0	8.66	5.19	0.0
CD27+IgM-	3.78	0.0	1.86	4.43	0.0
CD27-IgM+IgD+CD45RB+	2.02	18.36	10.31	4.88	0.0
Germinal centre	1.66	0.0	17.83	1.0	118.9
Naive	1.0	32.7	10.25	1.15	0.0

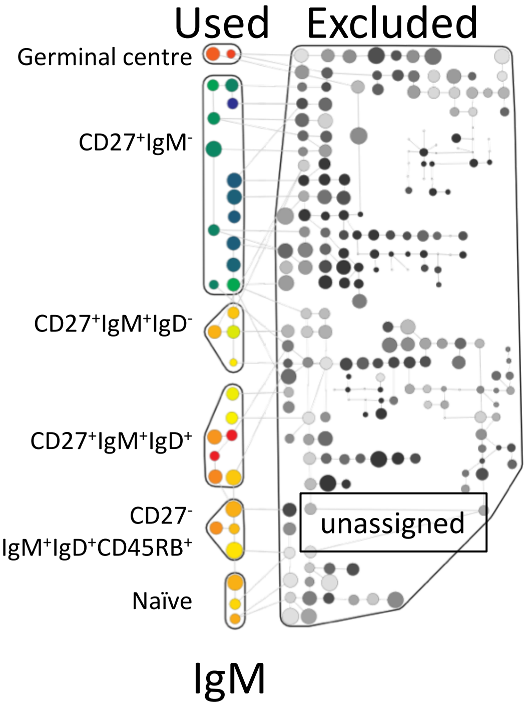

SPADE pixel events representing B cell subsets were exported from Cytobank and converted in MATLAB back into pixels. The B Cell subset pixels were mapped back to the region of interest in the original scanned figure and pseudocoloured in MCD viewer.

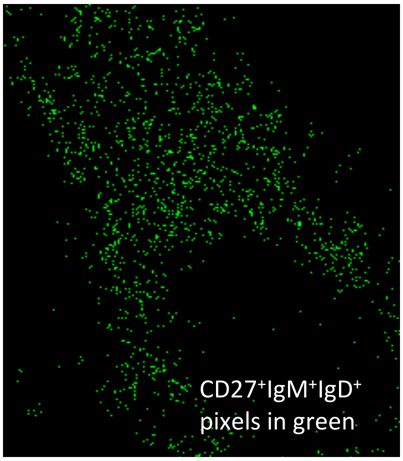

***Supplementary Figure 5**: Work flow for processing of image mass cytometry for representation of the distribution B cell subsets as pseudocoloured pixels.*

Supplementary Figure 6

a

	CD45RB mean expression in tissue defined subsets	CD24 mean expression in tissue defined subsets
Marginal zone ($CD19^+CD20^+CD27^+IgM^+IgD^+$)	6.44	4.54
IgM only ($CD19^+CD20^+CD27^+IgM^+IgD^-$)	5.50	3.80
Class switched memory ($CD19^+CD20^+CD27^+IgM^-IgD^-$)	5.80	2.03
Germinal centre	1.63	2.74
Naive	1.66	1.27

b

c

d

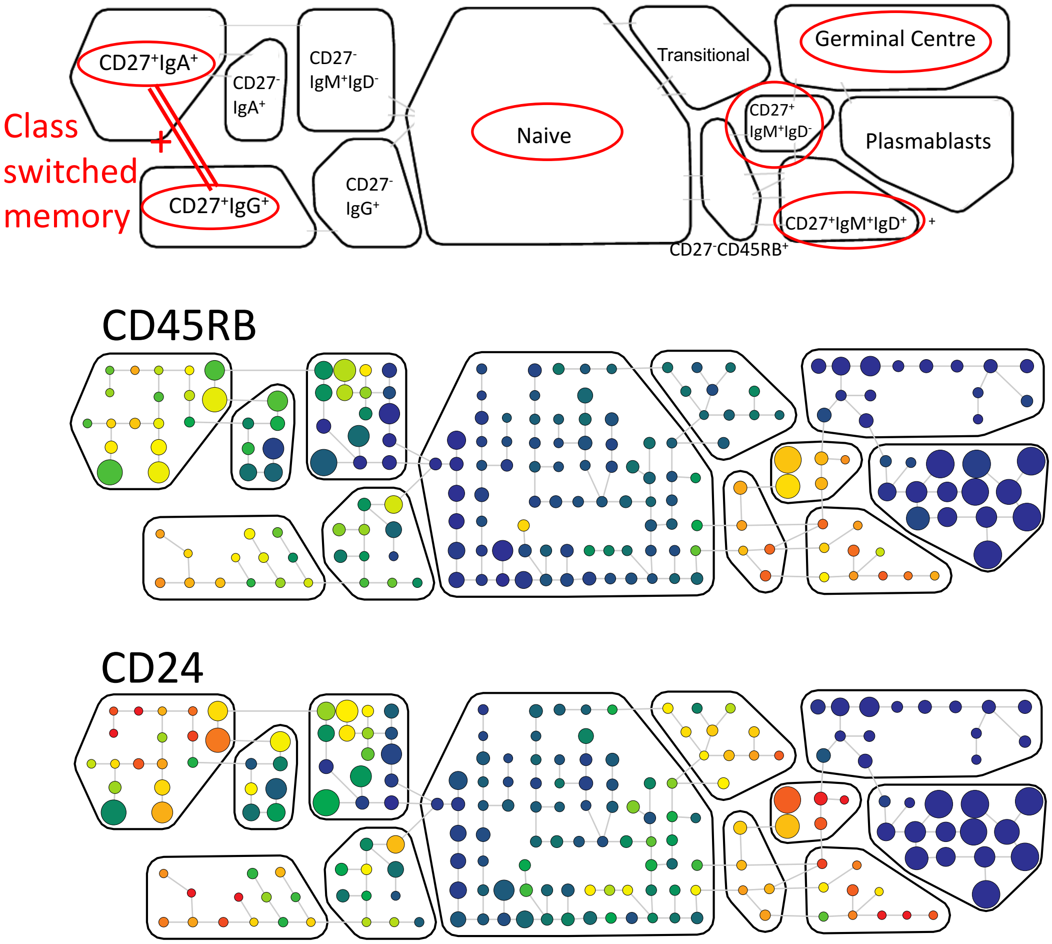

Supplementary Figure 6: Cross-validation of B cell subset classifications between imaging mass cytometry and mass cytometry.

a. For imaging mass cytometry: mean expression values of CD45RB and CD24 in pixels assigned to B cell subsets as described in Supplementary Fig.7. Importantly CD45RB and CD24 were not used in the subset designations by imaging mass cytometry. b. Key to the identification of the B cell subsets in the mass cytometry SPADE diagrams in c and d. c and d. For mass cytometry: expression of CD45RB and CD24 respectively, across B cell subsets in human GALT. Broad agreement was observed expression of CD45RB and CD24 by B cell subsets identified by imaging mass cytometry and mass cytometry.

Supplementary Figure 7

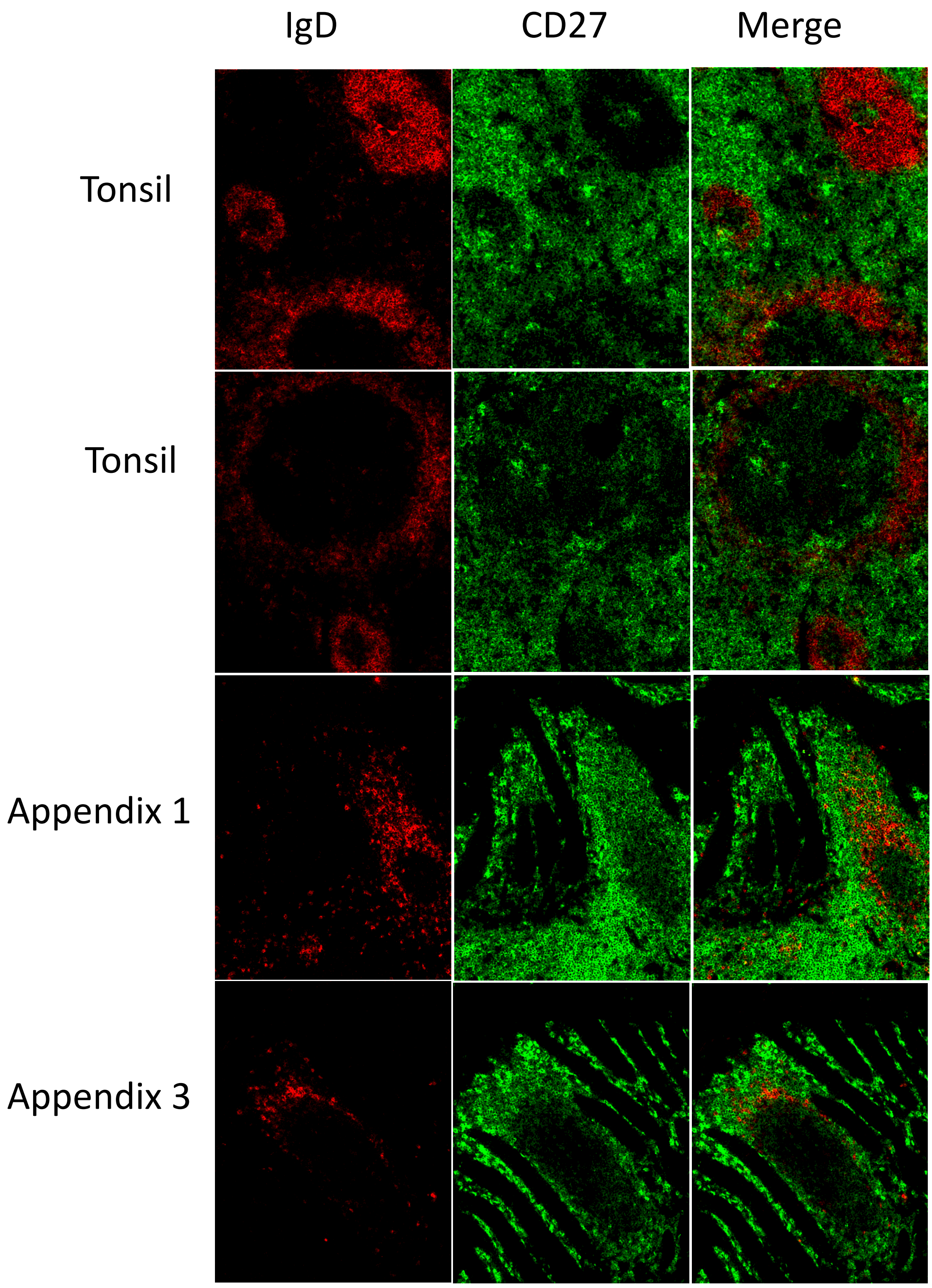

Supplementary Figure 7: Some samples of appendix contain few naïve B cells when visualised by imaging mass cytometry.

Whereas the GC of tonsil are consistently surrounded by a broad zone naïve B cells forming the mantle zone, there is often a sparsity of naïve B cells in the equivalent microenvironment in GALT. In appendix 1 and 3 in this study many IgD^{+} cells are CD27 expressing marginal zone B cells.

Supplementary Figure 8

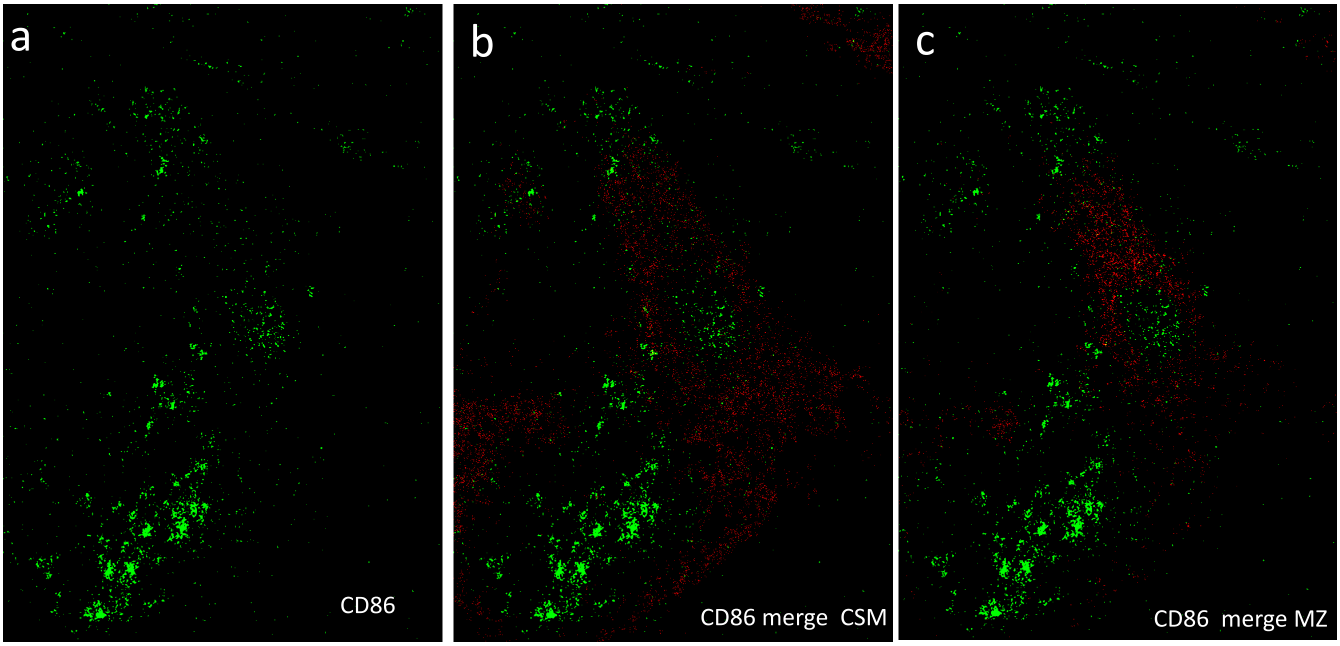

Supplementary Figure 8: Colocalisation of CD86 with B cells was not detected outside the GC by imaging mass cytometry.
a. Distribution of CD86 staining in human appendix detected by imaging mass cytometry pseudocoloured green. b. CD86 staining did not co-localise with the profile $CD19^{+}CD20^{+}CD27^{+}IgM^{-}IgD^{-}$ (representative of class switched memory cells) or c. with pixels with the profile $CD19^{+}CD20^{+}CD27^{+}IgM^{+}IgD^{+}$ (representative of marginal zone B cells).

Supplementary Figure 9

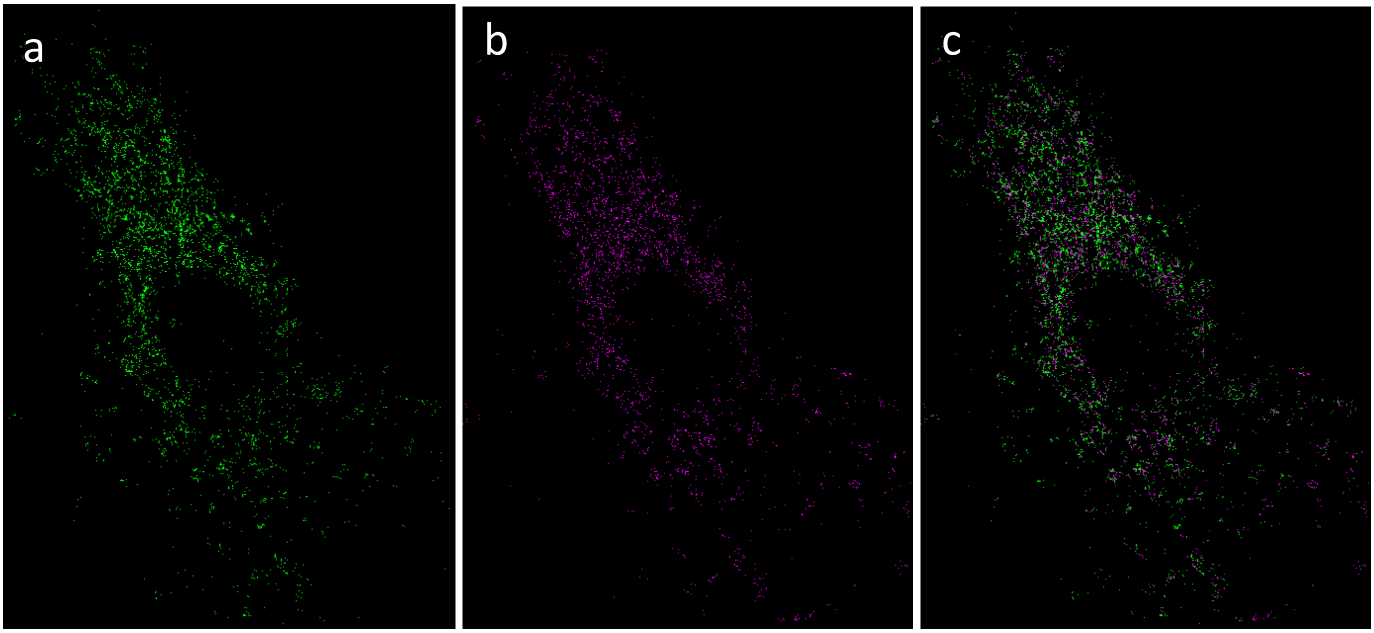

Supplementary Figure 9: Marginal zone B cells and their CD27-CD45RB+ precursors occupy the same areas of human appendix tissues by imaging mass cytometry
a. Pixels sorted by the criteria $CD19^{+}CD20^{+}CD27^{+}IgM^{+}IgD^{+}$ (representative of marginal zone) were pseudocoloured green. b. Pixels sorted by the criteria $CD19^{+}CD20^{+}CD27^{-}IgM^{+}IgD^{+}CD45RB^{+}$ (representative of CD45RB marginal zone precursor cells) were pseudocoloured magenta. c. Overlay of A and B illustrates that pixels representing these 2 subsets occupy the same areas of tissues.

Supplementary Figure 10

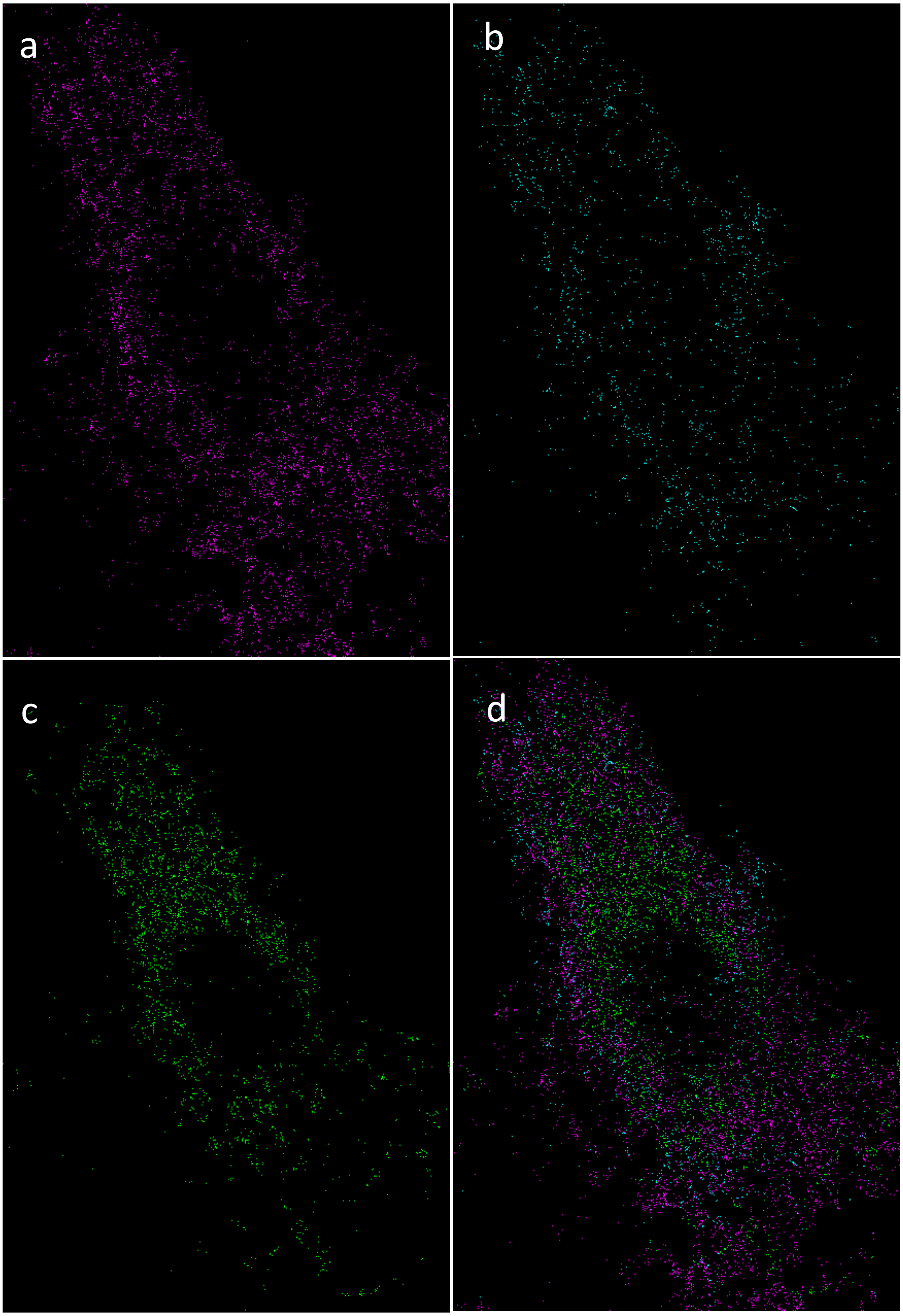

Supplementary Figure 10: IgM-only cells can locate in areas predominantly occupied by either marginal zone B cells or class switched memory B cells.

a. Pixels sorted by the criteria $CD19^{+}CD20^{+}CD27^{+}IgM^{-}IgD^{-}$ (representative of class switched memory B cells) were pseudocoloured magenta. b. Pixels sorted by the criteria $CD19^{+}CD20^{+}CD27+IgM^{+}IgD^{-}$ (representative of IgM-only B cells) were pseudocoloured cyan. c. Pixels sorted by the criteria $CD19^{+}CD20^{+}CD27^{+}IgM^{+}IgD^{+}$ (representative of marginal zone B cells) were pseudocoloured green. Overlay of a,b and c illustrates that whereas class switched memory and marginal zone B cells tend to have different distributions, pixels representing IgM only B cells can occupy the same areas of tissues as either class switched memory and marginal zone B cells.

Supplementary Figure 11

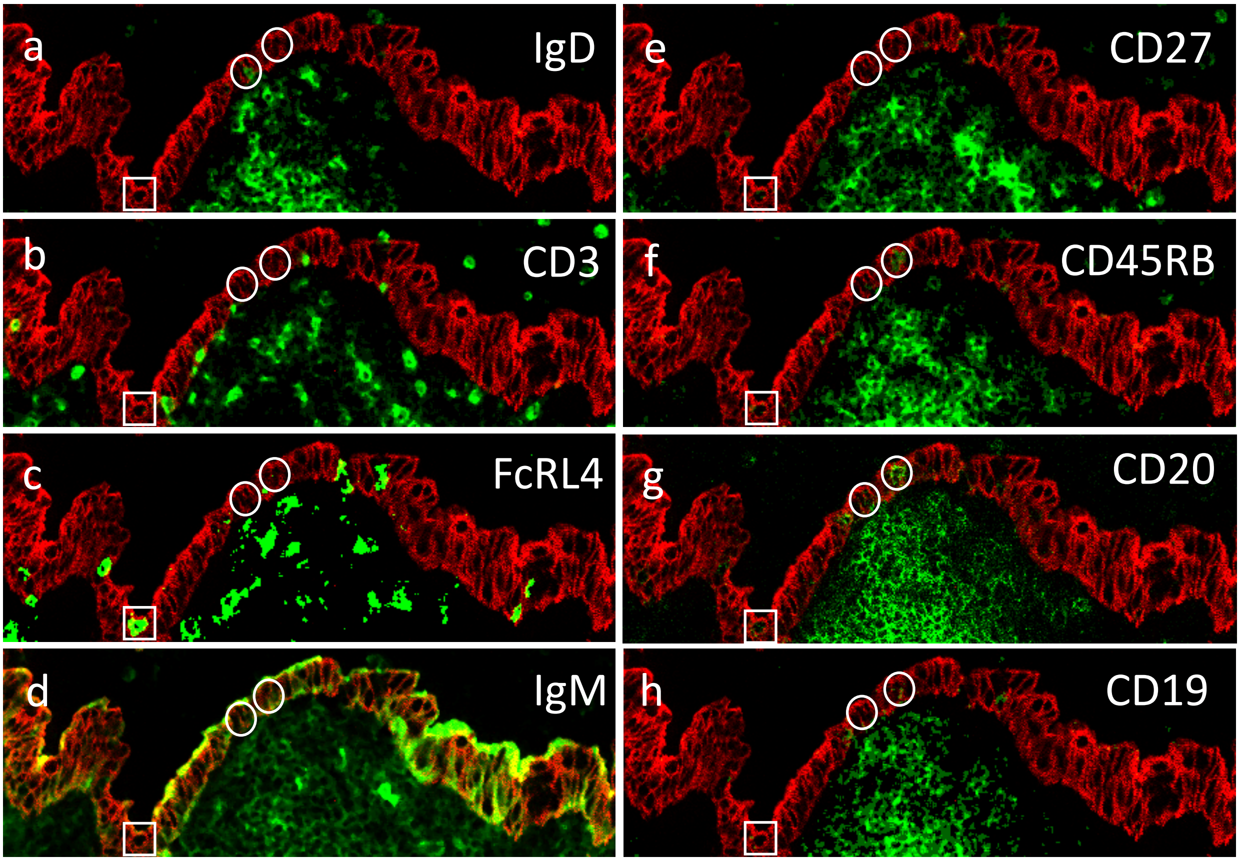

Supplementary Figure 11. B cells in human follicle associated epithelium are phenotypically varied when visualised by imaging mass cytometry.
Visualisation of antibody binding to cytokeratin of follicle-associated epithelium in red in all images and in a-h to IgD, CD3, FcRL4, IgM, CD27, CD45RB, CD20 and CD19 respectively in appendix 2 in green. Comparison of images reveals phenotypic variability in intraepithelial B cells. Circles and the rectangle are included to aid comparisons between images.

Supplementary Figure 12

a

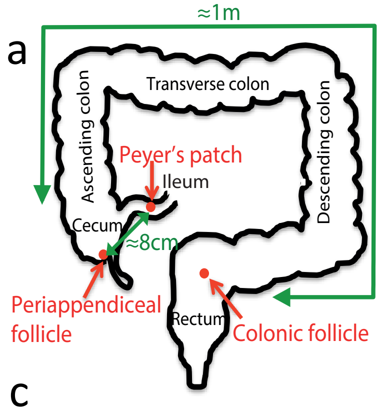

b

Experiment	Total	M:F	Age (average and range)
B cell subsets by flow cytometry	19	10:9	45.1 (27-77)
IgA,IgG and IgM expression by flow cytometry	9	5:4	41.2 (26-61)
Cell cyTOF	8	3:5	52.9 (27-76)
RT-PCR for AID expression	5	2:3	44 (27-55)
High throughput IGV gene sequencing	4	1:1	42 (27-55)

c

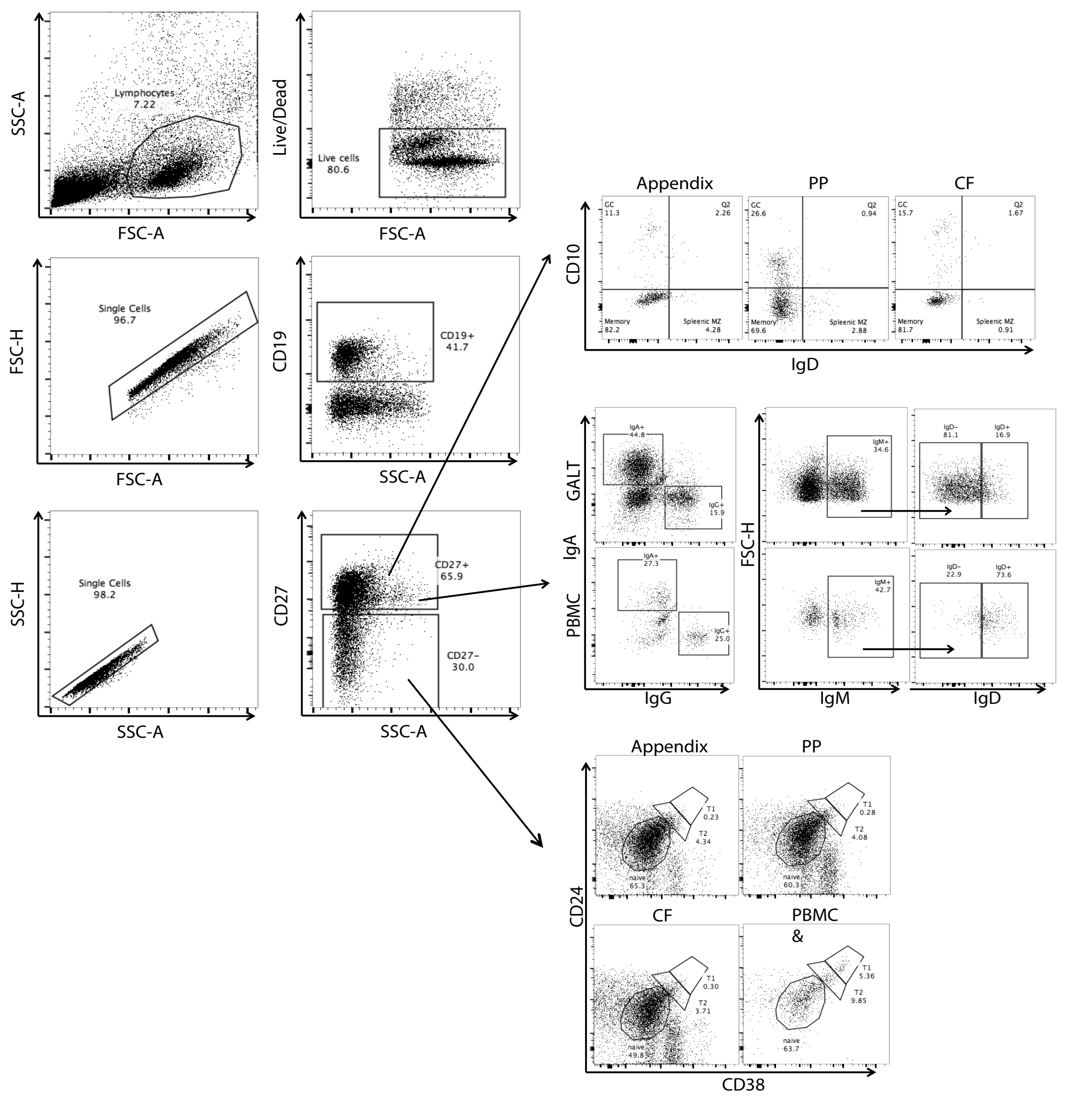

Supplementary Figure 12: Gating strategies for characterisation of B cells from GALT.
a. Biopsies of Peyer's patch, periappendiceal follicle and colonic follicle were sampled from three sites of human gut. The approximate distances between Peyer's patch biopsy and periappendiceal follicle or colonic follicle biopsies are 8cm and 1 meter respectively. b. Summary of age and gender of patients donating tissues for analysis of B cell composition, isotype expression, next generation sequencingand RT-PCR experiments. c. Flow cytometry gating strategy. For B cell composition, live single cells from GALT, spleen and PBMC were gated $CD19^+CD27^+CD10^+IgD^-$ (GC), $CD19^+CD27^+CD10^-IgD^+$ (splenic MZ), $CD19^+CD27^+CD10^-IgD^-$ (memory), $CD19^+CD27^-$ CD24hiCD38hi (T1), $CD19^+CD27^-CD24^+CD38^+$ (T2), and $CD19^+CD27^-CD24^-CD38^-$ (naïve). For immunoglobulin isotype expression, $CD19^+CD27^+$ B cells from GALT and PBMC were gated IgA^+IgG^-, IgA^-IgG^+, IgM^+IgD^+, and IgM^+IgD^-. Stained cells were detected by Fortessa (BD) and analyzed using Flowjo software.

Supplementary Figure 13

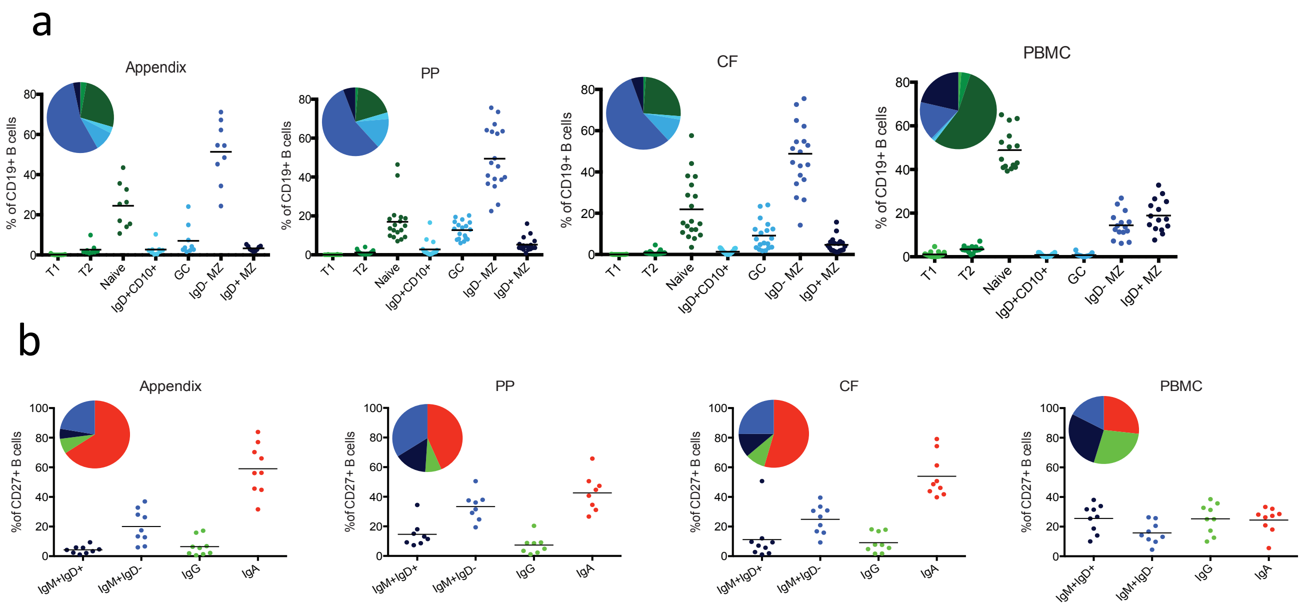

Supplementary Figure 13: a. Scatter plots and pie charts to represent B cell subset composition of GALT in appendix, PP and CF and B. blood (PBMC) from the donors of GALT and tonsil (for gating see Supplementary Figure14). b. Scatter plots and pie charts where color represents the distribution of Ig isotypes expressed by CD19^{+}, CD27^{+} B cells from matched GALT from appendix, PP colonic follicle CF and blood (see Supplementary Figure14).

Supplementary Figure 14

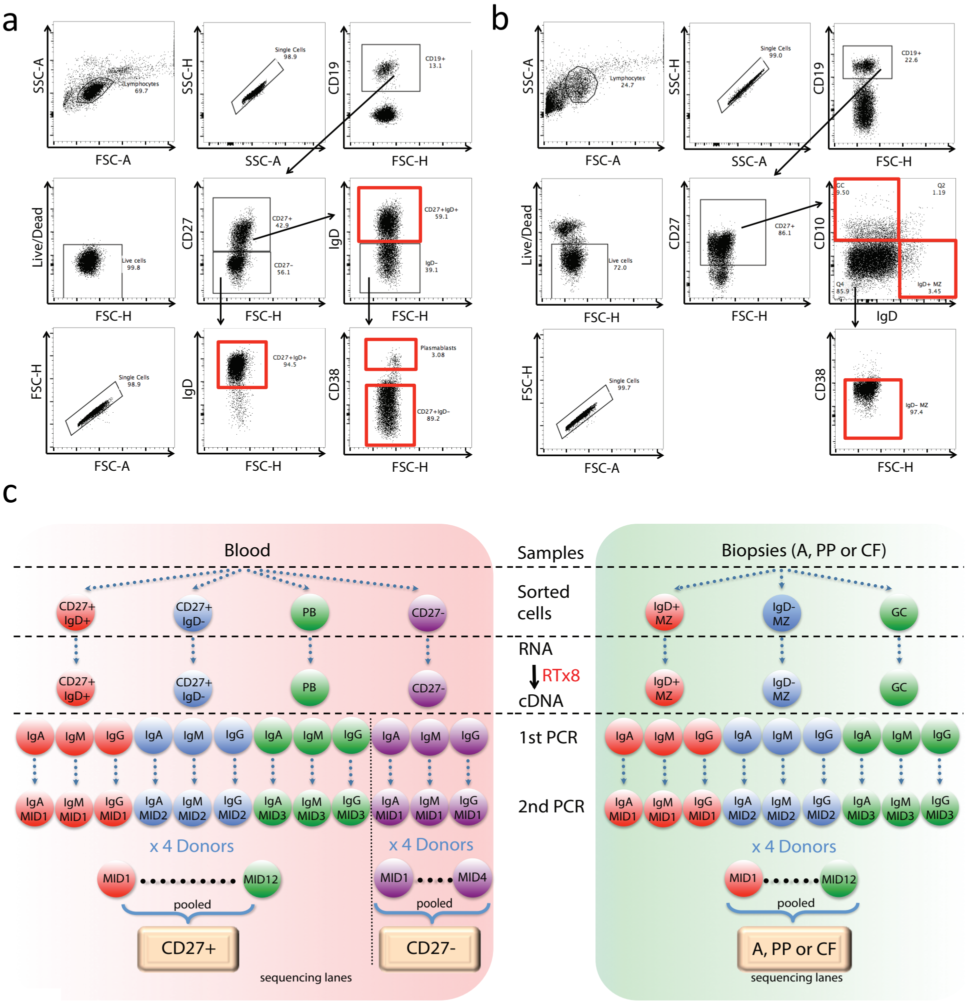

d

	Appendix				PP				CF			
	Donor 1	Donor 2	Donor 3	Donor 4	Donor 1	Donor 2	Donor 3	Donor 4	Donor 1	Donor 2	Donor 3	Donor 4
GC	887	17902	2187	7097	3666	15975	11549	3317	279	15916	5228	974
IgD+ MZ	454	1398	949	3557	224	1736	2239	2761	84	5532	2270	1412
IgD- MZ	13741	13009	21397	29882	5719	31942	148901	15917	3904	20006	28799	28046

	PBMC			
	Donor 1	Donor 2	Donor 3	Donor 4
CD27+IgD+	20862	142706	84718	206540
CD27+IgD-	23086	205845	269496	389794
Plasmablasts	8165	11945	11492	14349
CD27-IgD+	60776	483898	316373	296366

Supplementary Figure 14. a. FACS strategy for isolation of $CD27^{+}IgD^{+}$, $CD27^{+}IgD^{-}$, $CD27^{-}IgD^{+}$ B cells and plasmablasts from PBMC based by their expression of CD19, CD27, IgD, and CD38. b. FACS strategy for isolation of germinal center (GC), $CD27^{+}IgD^{+}$, $CD27^{+}IgD^{-}$ B cells from gut biopsies based on their expression of CD19, CD27, IgD, CD10 and CD38. c. Experimental strategy for high throughput sequencing for B cells sorted from PBMC and gut biopsies. RNA was isolated from each sorted cell subset, then synthesized to cDNA individually. Reverse transcription was repeated 8 times for every sample to increase the sampling efficiency. IgA, IgM and IgG PCR was performed separately in the PCR1 for each sample. MID-tagged primers were used in PCR2. After purification, all PCR2 products of $CD27^{+}IgD^{+}$, $CD27^{+}IgD^{-}$ B cells and plasmablasts sorted from PBMC of 4 donors were pooled into one sequencing lane named $CD27^{+}$; all 2nd round PCR products of $CD27^{-}$ B cells of PBMC from 4 donors were pooled into one sequencing lane named $CD27^{-}$; all 2nd round PCR products of $CD27^{+}IgD^{+}$, $CD27^{+}IgD^{-}$ and GC B cells sorted from different gut biopsies (appendix, Peyer's patch or colonic follicle) of 4 donors were pooled into three different sequencing lanes (appendix, Peyer's patch, and colonic follicle) respectively. d. Numbers of sorted cells from appendix, Peyer's patch, colonic follicle and PBMC of 4 donors.

Supplementary Figure 15

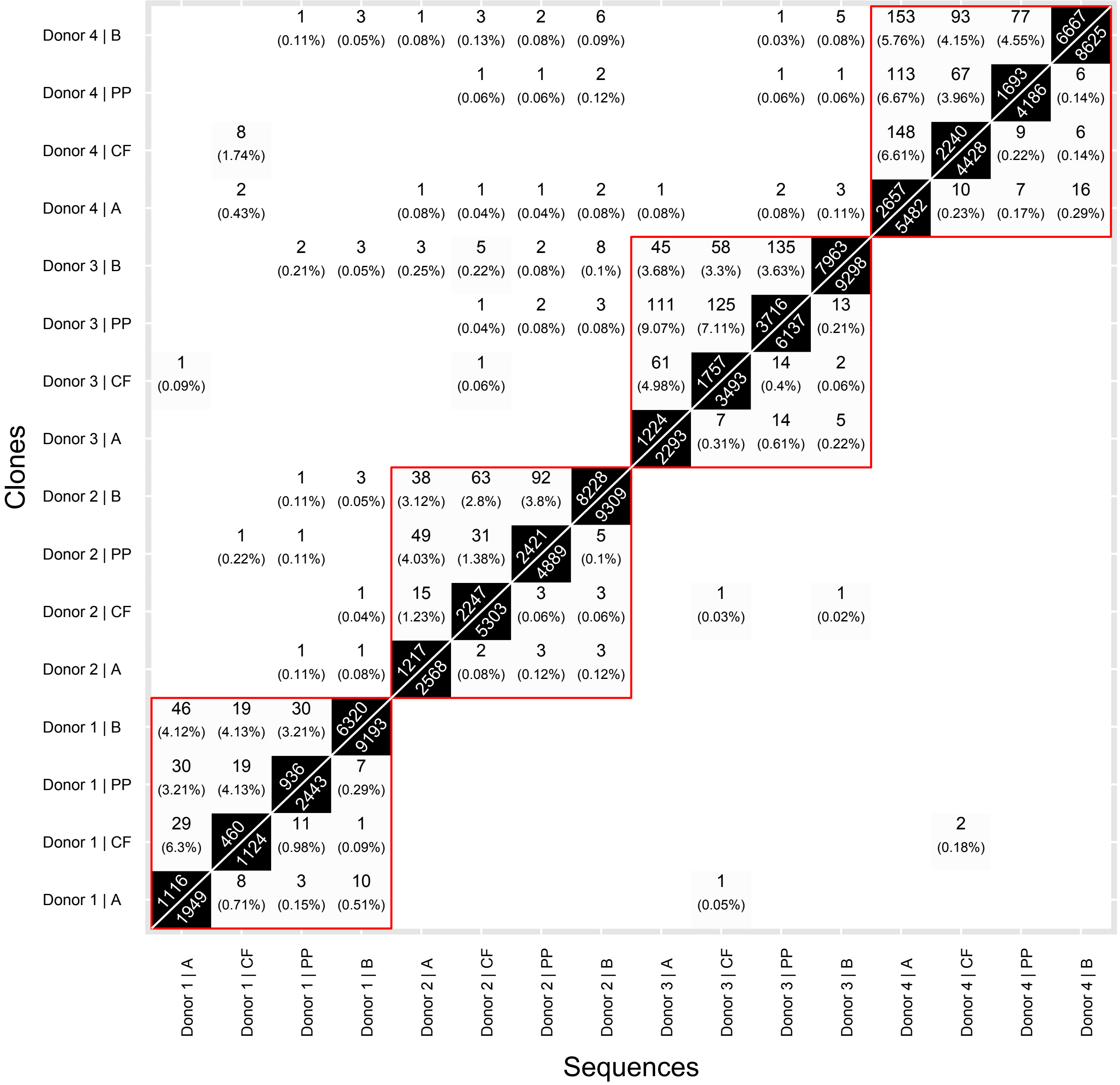

Supplementary Figure 15: The percentage of clones (top triangle) and unique sequences (bottom triangle) shared between donors and across tissue sites. Numbers on the diagonal indicate the total number of clones or unique sequences per site, with the off-diagonal numbers indicating the number of shared clones or sequences (percentage of the smaller number in parenthesis). The red boxes identify individuals and the data within these boxes express the extent of sharing of clones and unique sequences between tissue sites.

Supplementary Figure 16

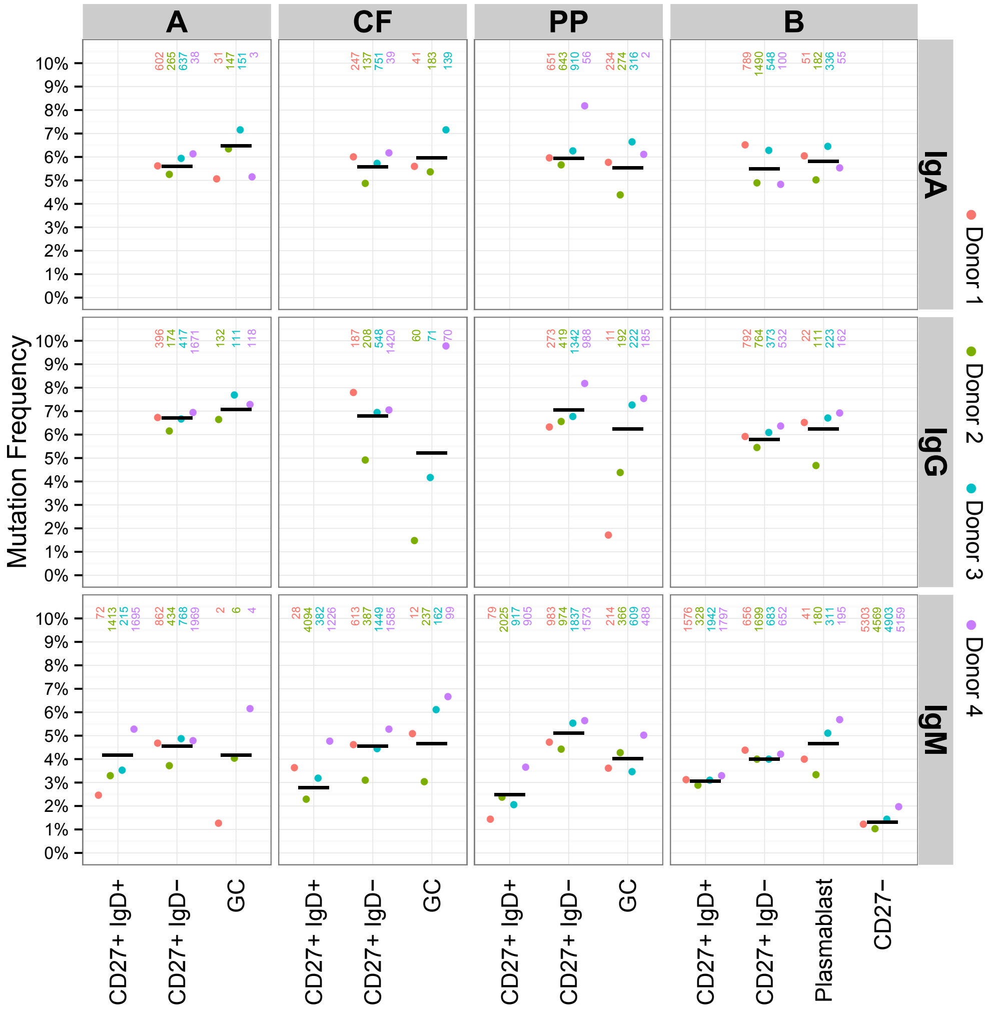

Supplementary Figure 16: Frequency of somatic mutations in IGHV associated with each isotype, in each B cell subset, isolated from each site from donors 1-4.

Supplementary Figure 17

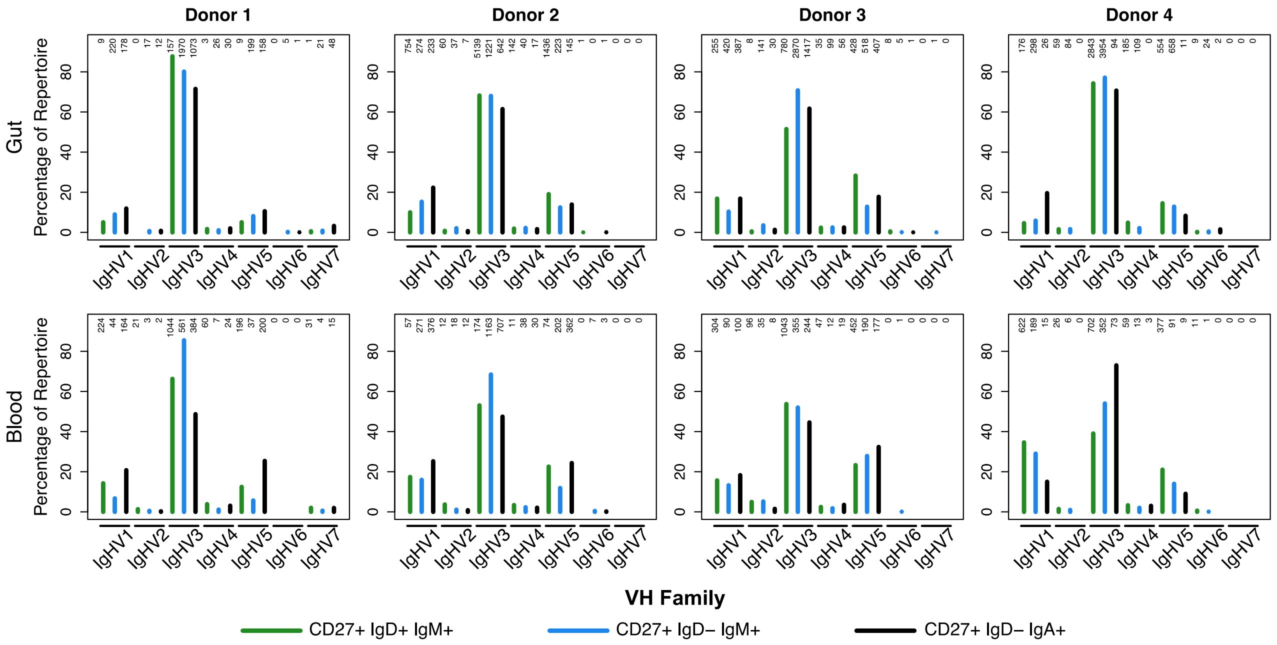

Supplementary Figure 17: IGHV family gene segments as a percentage of the repertoire used by CD27^{+}IgM^{+}IgD^{+}, CD27^{+}IgM^{+}IgD^{-}, CD27^{+}IgA^{+} sequences in gut and blood for donors 1-4.

Supplementary Figure 18

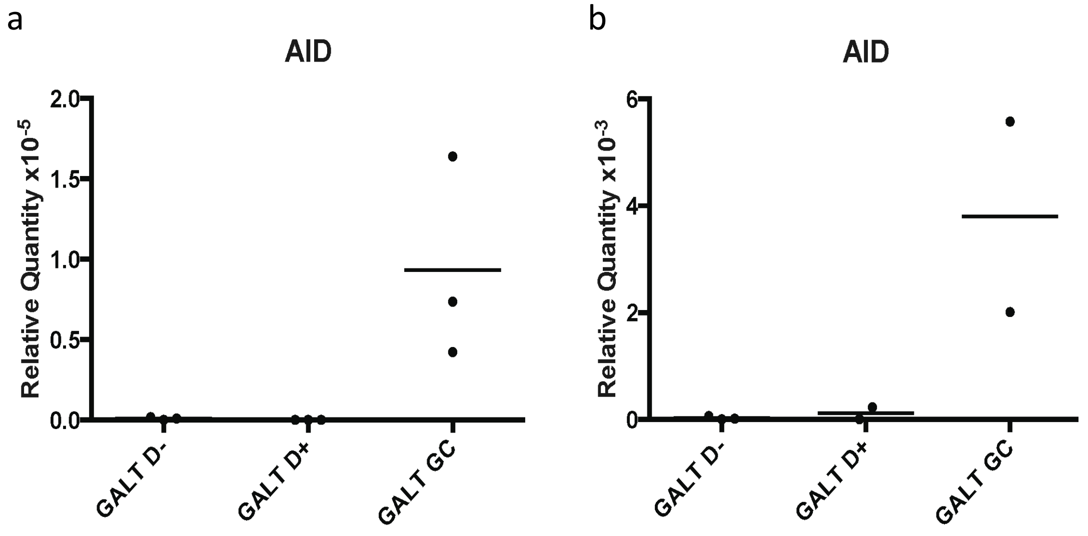

Supplementary Figure 18: Analysis of AID expression. By B cells from GALT. a and b. Relative quantity of AID standardized to 18s in CD27+IgD+, CD27+IgD- and GC B cells of 3 biopsies from healthy donors. For a. the AID primer probe was designed using the Roche Universal probe library system (AID F 1:ggactttggttatcttcgcaat, AID R 1: gtcgggcacagtcgtagc). b. Relative quantity of AID standardized to 18s in CD27+IgD+, CD27+IgD- and GC B cells of 2 isolates from additional healthy donors. For b the AID primer probe mix (Hs00757808_m1 AICDA) was purchased from Applied Biosystems.

Supplementary Figure 19

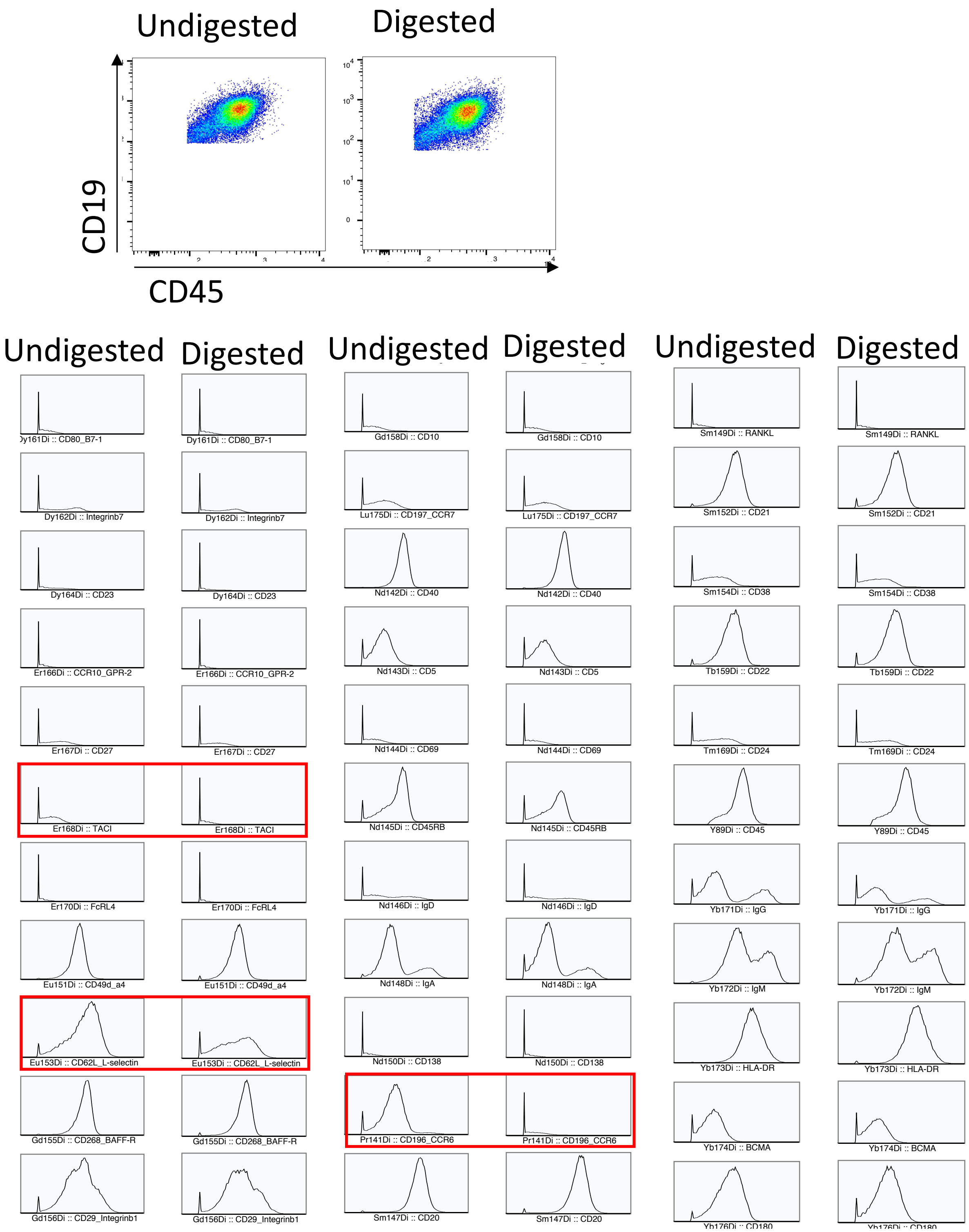

Supplementary Figure 19: Affect of mild collagenase digestion as used for GALT lymphocyte isolation on detection of antigens by liquid mass cytometry.
PBMC were either digested or not with collagenase according to the protocol used for isolation of GALT cells and sample staining compared by cyTOF. Although digestion left most antigens unaltered, there were reductions in detection of TACI, CD62L and CCR6 by this processing method.

Supplementary Figure 20

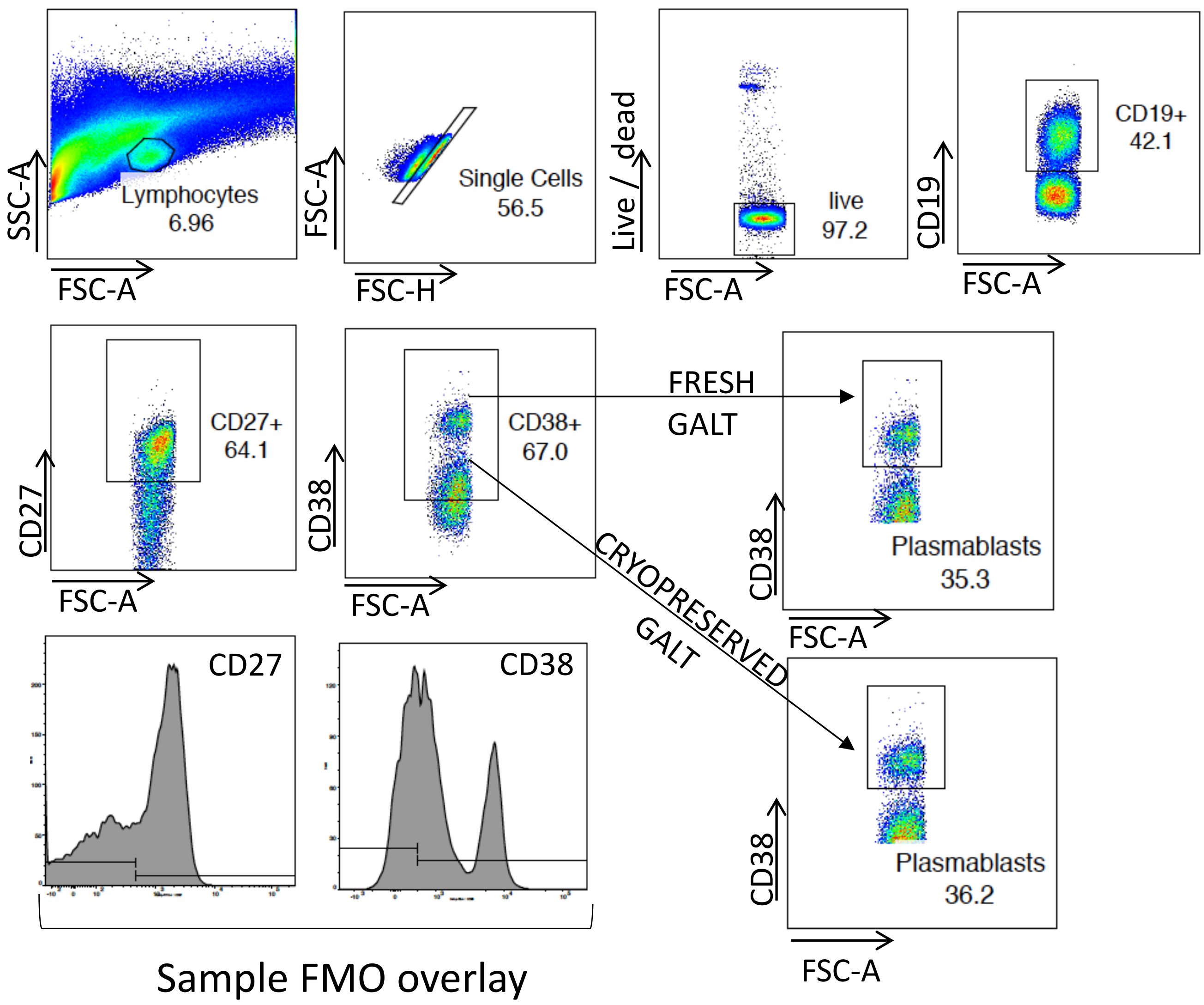

Supplementary Figure 20: Effect of cryopreservation on plasmablast frequencies. Since GALT B cells had a relatively large plasmablast component evident in the SPADE plots and because this was the only one of the 3 populations to have been processed fresh, we tested if cryopreservation may have selectively depleted the tonsil and spleen samples of plasmablasts. Data above that details the gating strategy by flow shows that this was not the case.

Supplementary Figure 21

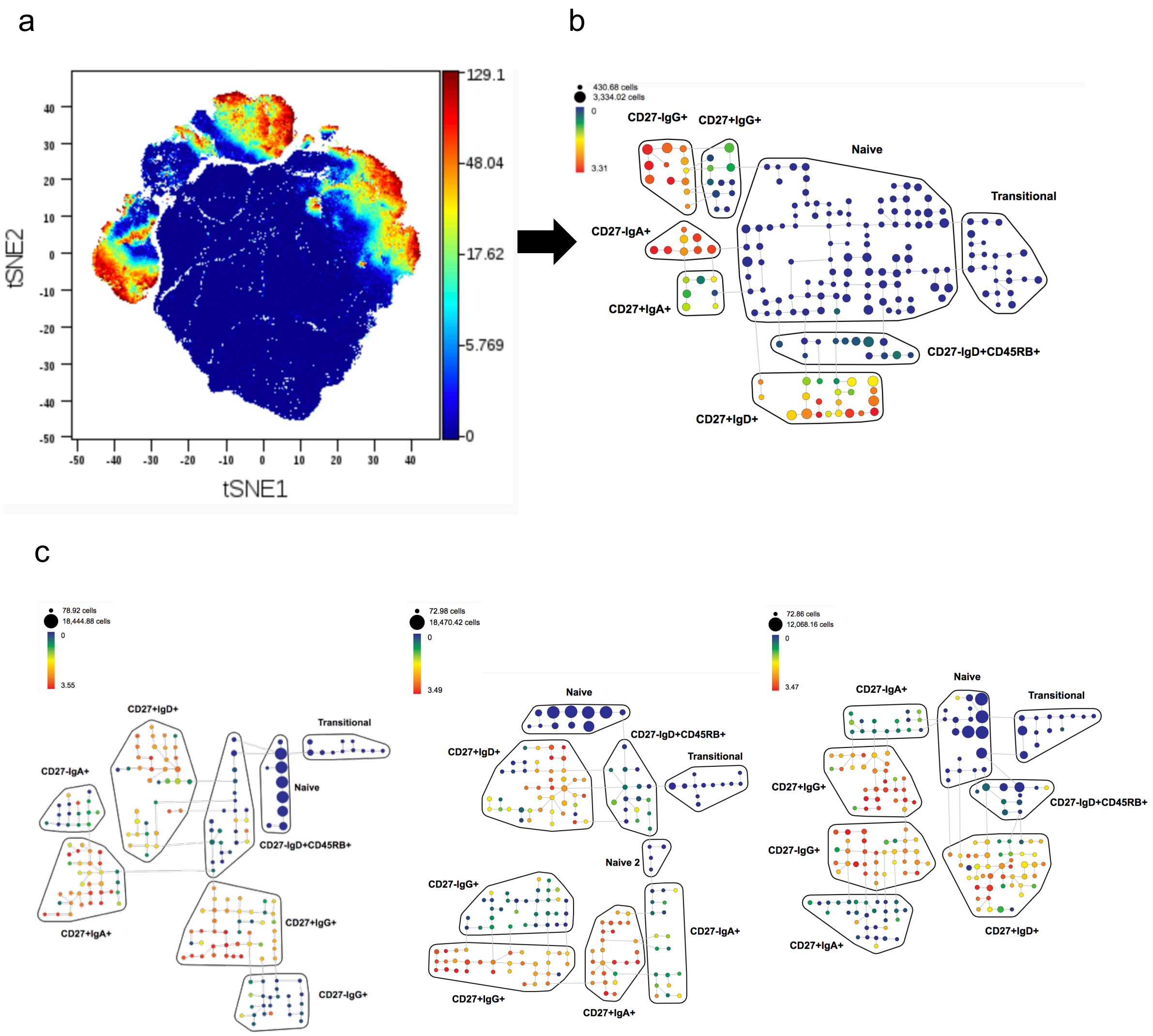

Supplementary Figure 21: Comparison of SPADE and SPADE on viSNE in Cytobank. a. viSNE plot depicting CD27 blood B cells concatenated from 5 female healthy controls. b. SPADE plot depicting CD27 expression generated using tSNE1 and tSNE2 coordinates from the viSNE plot in part a. c. In order to assess the reproducibility of SPADE as a clustering tool alone, three successive SPADE analyses were conducted using the same dataset and clustering parameters as part a. The plots were taken from successive runs and depict CD27 expression.

Supplementary Figure 22

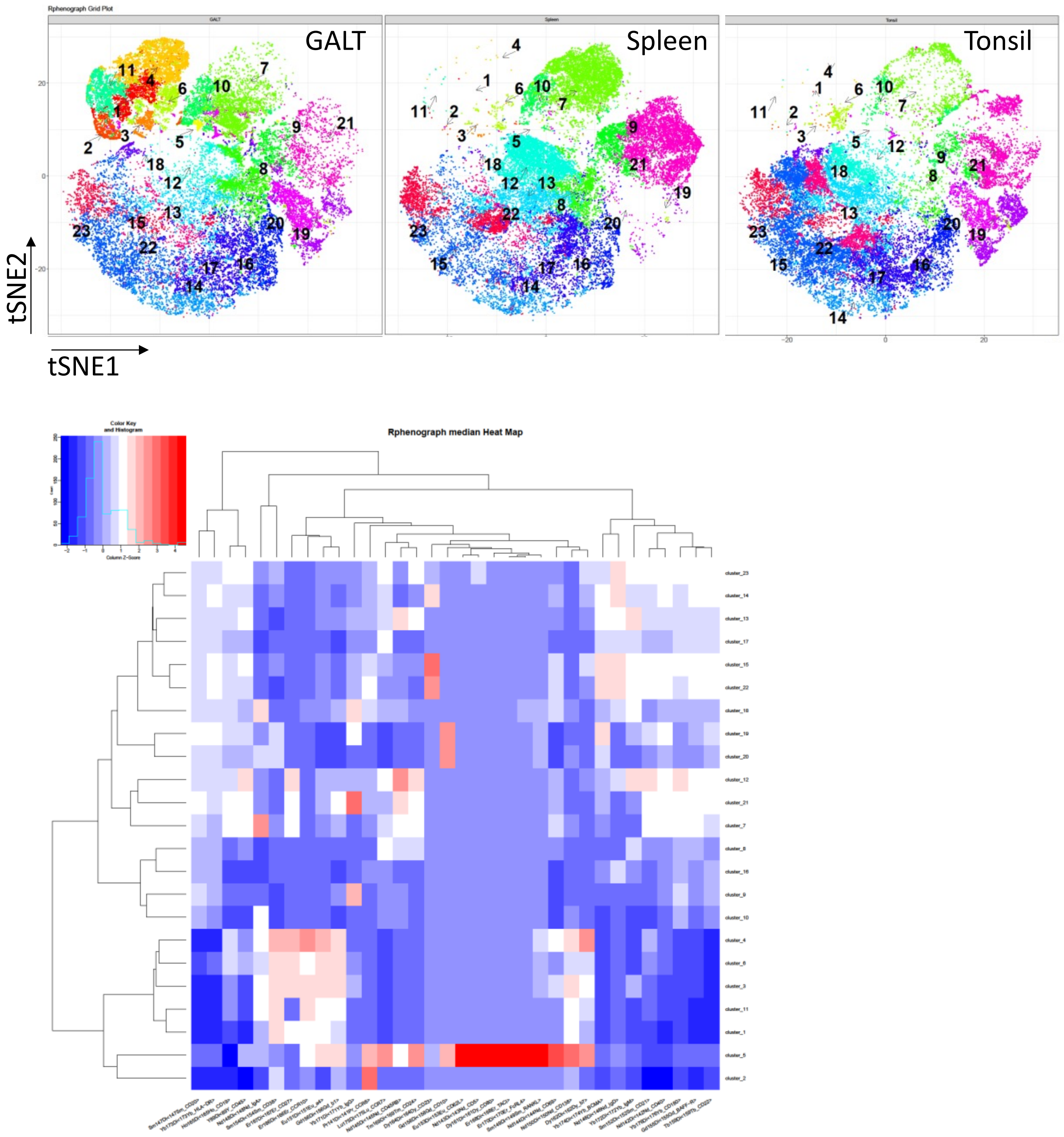

Supplementary Figure 22: An example of use of an unsupervised method to analyse mass cytometry data. Use of PhenoGraph[1] to analyse mass cytometry data. -tSNE using CD27, CD10, CD24, IgA, IgG, IgD, IgM, HLA-DR and CD38. PhenoGraph enabled unsupervised identification of clusters and differences between tissues. However the clusters identified did not relate easily to the existing literature or other data in the manuscript

Supplementary Figure 23

a

	A	CF	PP	B1	B2
**Raw reads**	124664	104360	109134	110600	91920
**QC Filtering**	70235	49834	59946	57403	37276
**Duplicate Removal**	40437	28044	32400	36567	32296
**Germline Alignment**	39183	26992	31290	34436	31577
**QC'd and Productive**	28042	19887	23588	25151	22779

b

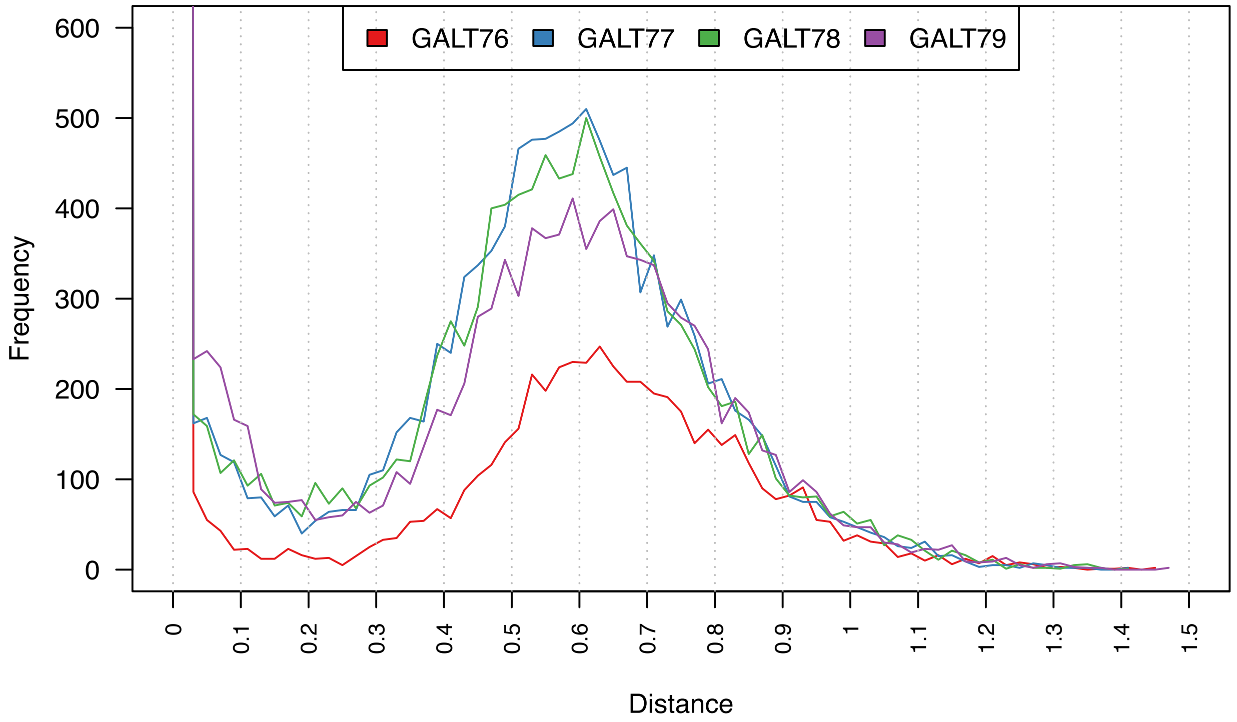

Supplementary Figure 23: Quality control for NGS reads

a. Quality control pipeline for processing of raw reads. b. Distance between sequences was measured as the number of nucleotide differences in the CDR3, with each difference weighted by the human S5F model of somatic hypermutation targeting and substitution (Yaari et al., 2013) as previously described (Stern et al., 2014). This distance was normalized by dividing by the CDR3 length. The threshold of 0.2 was chosen by manual inspection of the distance-to-nearest plots generated for each individual.

Supplementary Table 1

Target	Metal	Clone	Working dilution	Supplier
BCMA	174Yb	19F2	1:100	Biolegend
CCR10 (GPR-2)	166Er	314305	1:100	R&D Systems
CD10	158Gd	HI10a	1:100	Fluidigm
CD138	150Nd	DL-101	1:100	Fluidigm
CD180	176Yb	MHR73-11	1:100	Biolegend
CD19	165Ho	HIB19	1:100	Fluidigm
CD196 (CCR6)	141Pr	11A9	1:100	Fluidigm
CD197 (CCR7)	175Lu	G043H7	1:100	Biolegend
CD20	147Sm	2H7	1:100	Fluidigm
CD21	152Sm	BL13	1:100	Fluidigm
CD22	159Tb	HIB22	1:100	Fluidigm
CD23	164Dy	EBVCS-5	1:100	Fluidigm
CD24	169Tm	ML5	1:100	Fluidigm
CD268 (BAFF-R)	155Gd	11C1	1:100	Fluidigm
CD27	167Er	O323	1:100	Fluidigm
CD29 (integrin β1)	156Gd	TS2/16	1:100	Fluidigm
CD38	154Sm	HIT2	1:100	Biolegend
CD40	142Nd	5C3	1:100	Fluidigm
CD45	89Y	HI30	1:100	Fluidigm
CD45RB	145Nd	MEM-55	1:100	Fluidigm
CD49d (α4)	151Eu	9F10	1:100	Biolegend
CD5	143Nd	UCHT2	1:100	Fluidigm
CD62L (L-selectin)	153Eu	DREG-56	1:100	Fluidigm
CD69	144Nd	FN50	1:100	Fluidigm
CD80 (B7-1)	161Dy	2D10.4	1:100	Fluidigm
FcRL4	170Er	413D12	1:100	Biolegend
HLA-DR	173Yb	L243	1:100	Fluidigm
IgA	148Nd	Polyclonal	1:100	Fluidigm
IgD	146Nd	IA6-2	1:100	Fluidigm
IgG	171Yb	Polyclonal	1:100	Fluidigm
IgM	172Yb	MHM-88	1:100	Fluidigm
Integrin β7	162Dy	FIB504	1:100	Fluidigm
RANKL	149Sm	MIH24	1:100	Biolegend
TACI	168Er	1A1	1:100	Biolegend
CD3	160Gd	UCHT1	1:100	Bilegend
CD14	160Gd	M5E2	1:100	Fluidigm

Supplementary Table 1: Details of antibodies used for liquid mass cytometry. Targets, metal tags, clone names and supplier of antibodies used for liquid mass cytometry

Supplementary Table 2

Target	Metal	Clone	Working dilution	Supplier
CD19	176Yb	HIB19	1 in 100	Fluidigm
CD20	161Dy	2H7	1 in 250	Fluidigm
CD10	158Gd	HI10a	1 in 50	Fluidigm
CD24	169Tm	ML5	1 in 100	Fluidigm
CD45RB	145Nd	MEM55	1 in 50	Biolegend
CD38	167Er	HIT2	1 in 100	Biolegend
IgM	172Yb	MHM88	1 in 50	Fluidigm
IgD	159Tb	IgD26	1 in 50 gut<br>1 in 100 tonsil	Miltenyi
CD86	165Ho	IT2.2	1 in 50	Biolegend
CD27	155Gd	0323	1 in 100	Fluidigm
FcRL4	170Er	413D12	1 in 50	Biolegend
CD45	89Y	HI30	1 in 200	Fluidigm
CD3	154Sm	UCHT1	1 in 100	Fluidigm
PanKeratin	148Nd	C11	1 in 1500	Fluidigm
DNA	191Ir	N/A	0.5uM	Fluidigm

Supplementary Table 2**: **Details of antibodies used for tissue mass cytometry. Targets, metal tags, clone names and supplier of antibodies used for tissue mass cytometry

Supplementary Table 3

a

PCR1 primer name	PCR1 primer sequences
IGHV1	CCTCAGTGAAGGTCTCCTGCAAGG
IGHV2	TCCTGCGCTGGTGAAACCCACACA
IGHV3	GGTCCCTGAGACTCTCCTGTGCA
IGHV4	TCGGAGACCCTGTCCCTCACCTGC
IGHV5	CAGTCTGGAGCAGAGGTGAAA
IGHV6	CCTGTGCCATCTCCGGGGACAGTG
CHA"	GGCTCCTGGGGGAAGAAGCC
CHG"	GAGTTCCACGACACCGTCAC
CHM"	GGGGAATTCTCACAGGAGAC

b

PCR2 multiplex identifiers		PCR2 gene specific sequences	
**Barcodes**	**Sequences (5'-3')**	**Primer names**	**Sequences (5'-3')**
MID 1	acgagtgcgt	IGHV1	CCTCAGTGAAGGTCTCCTGCAAGG
MID 2	acgctcgaca	IGHV2	TCCTGCGCTGGTGAAACCCACACA
MID 3	agacgcactc	IGHV3	GGTCCCTGAGACTCTCCTGTGCA
MID 4	agcactgtag	IGHV4	TCGGAGACCCTGTCCCTCACCTGC
MID 5	atcagacacg	IGHV5	CAGTCTGGAGCAGAGGTGAAA
MID 6	atatcgcgag	IGHV6	CCTGTGCCATCTCCGGGGACAGTG
MID 7	cgtgtctcta	CHA	GGAAGAAGCCCTGGACCAGGC
MID 8	ctcgcgtgtc	CHG	CACCGTCACCGGTTCGGGG
MID 9	tagtatcagc	CHM	CAGGAGACGAGGGGGAAAAGG
MID 10	tctctatgcg		
MID 11	tgatacgtct		
MID 12	tactgagcta		

Supplementary Table 3: PCR primers used for NGS

Details of a. first round (PCR1) and b. second round (PCR2) PCR primers[2]

References

1. Levine, J. H. *et al.* Data-Driven Phenotypic Dissection of AML Reveals Progenitor-like Cells that Correlate with Prognosis. *Cell* **162**, 184-197, doi:10.1016/j.cell.2015.05.047 (2015).
2. Wu, Y. C. et al. High-throughput immunoglobulin repertoire analysis distinguishes between human IgM memory and switched memory B-cell populations. Blood **116**, 1070-1078, doi:10.1182/blood-2010-03-275859 (2010)
